# Supplementary material for: Unravelling the operation of organic artificial neurons for neuromorphic bioelectronics
Source: Nat Commun. 2024 Jun 24;15:5350. doi: 10.1038/s41467-024-49668-1 (PMC11196688; doi:10.1038/s41467-024-49668-1)
Supplement: Supplementary file 1 — Supplementary Information [file 41467_2024_49668_MOESM1_ESM.pdf]

Supplementary Information

Unravelling the operation of organic artificial neurons for neuromorphic bioelectronics

Pietro Belleri[1], Judith Pons i Tarrés[2], Iain McCulloch[3], Paul W. M. Blom[2], Zsolt M. Kovács-Vajna[1], Paschalis Gkoupidenis[2,†], Fabrizio Torricelli[1,†]

[1] Department of Information Engineering, University of Brescia, via Branze 38, 25123 Brescia, Italy

[2] Max Planck Institute for Polymer Research, Ackermannweg 10, Mainz 55128, Germany

[3] Department of Chemistry, University of Oxford, 12 Mansfield Road, Oxford, United Kingdom

[†]Co-corresponding authors:

Dr. P. Gkoupidenis

Max Planck Institute for Polymer Research

Ackermannweg 10, Mainz 55128, Germany

E-mail: gkoupidenis@mpip-mainz.mpg.de

Prof. F. Torricelli

Department of Information Engineering

University of Brescia

Via Branze 38, 25123 Brescia, Italy.

E-mail: fabrizio.torricelli@unibs.it

Supplementary Table 1 | Comparison of state-of-art spiking artificial neurons. Spiking neurons fabricated with various materials, underling the key phenomena, the neuron circuit topology and the number of components. The comparison includes the ability of neurons to perform in-liquid ion-sensing and bio-interfacing, with biochemical-dependent spiking activities. Finally, spiking frequency, power consumption, and biophysical realism, viz. the ability to emulate realistically the electrical response of biological neurons, are provided. Abbreviations: Mott metal-insulator transition (Mott MIT), negative differential resistance (NDR), integrated circuit (IC), ring oscillator (RO), silicon on insulator (SOI), metal oxide semiconductor field effect transistor (MOSFET), transistor (T) capacitor (C), resistor (R), diode (D), not available (NA).

Material	Phenomena	Neuron topology	Components (#)	Neuron in-liquid ion-sensing and bio-interfacing	Spiking frequency (Hz)	Power consumption (µW)	Biophysical realism	Ref
Silicon	Electronic	Multi-component IC	Multi-T	No	~1-270	$75 \cdot 10^{-3}$	Yes	1
Silicon	Electronic	Multi-component IC	Multi-T	No	~200-900	$18 \cdot 10^{-3}$	Partially	2
Silicon	Electronic	SOI MOSFET	1T	No	~1-800	$\sim 22 \cdot 10^{-3}$	Yes	3
Silicon	Electronic	Bi-stable resistor	1T, 1C	No	~1-350	NA	Yes	4
Ferroelectric	Polarization	Transistor-like switching	1T	No	NA	NA	No	5
Ferromagnetic	Spintronic	Diode-like	1D	No	$250 \cdot 10^{6}$ $400 \cdot 10^{6}$	1	No	6
2D materials	Filament-based	Diode-like, switching	1D	No	NA	NA	No	7
Multi-component	Photonic	Multi-component	Multi-component	No	$1 \cdot 10^{3}$ $8.0 \cdot 10^{3}$	NA	Partially	8
Hybrid	Mott MIT	Diode-like, NDR	1D, 1R, 1C	No	$0.7 \cdot 10^{6}$ $2.7 \cdot 10^{6}$	NA	Partially	9
Metal-oxide	Mott MIT	Diode-like, NDR	2D, 2R, 2C-3C, 2 V_{int}	No	$\sim 5 \cdot 10^{3}$ $50 \cdot 10^{3}$	2	Yes	10
Metal-oxide	Mott MIT	Diode-like, NDR	1D, 1R, 1C	No	$0.4 \cdot 10^{6}$ $0.9 \cdot 10^{6}$	28	Partially	11
Metal-oxide	Vacancy-based	Diode-like	1D	No	NA	35	Partially	12
Metal-oxide	Mott MIT	Diode-like, NDR	2D, 3R, 3C	No	$\sim 8 \cdot 10^{3}$ $28 \cdot 10^{3}$	NA	Partially	13
Metal-oxide	Mott MIT	Diode-like, NDR	1D, 1R, 1C	No	$\sim 30 \cdot 10^{3}$ $70 \cdot 10^{3}$	NA	Yes	14
Metal-oxide	Mott MIT	Diode-like, NDR	1D	No	$\sim 2 \cdot 10^{6}$ $9 \cdot 10^{6}$	NA	Yes	15

Organic	Electronic	Diode-like, NDR	1D	No	$\sim 2\cdot 10^6$ $9\cdot 10^6$	NA	Yes	[16]
Organic	Electronic	Inverter-like, RO	9T, 2C, 1R	No	0.4-5	40	Partially	[17]
Organic	Iono-electronic	Inverter-like	5T, 2C	Partially	0.06-0.25	15	Partially	[18]
Organic	Iono-electronic	Transistor, inverter	4T, 1R, 1C	Yes	80	60	Yes	[19]
Organic	Iono-electronic	Transistor-like, NDR	2T, 2R, 1C	Yes	5-55	24	Yes	[20]

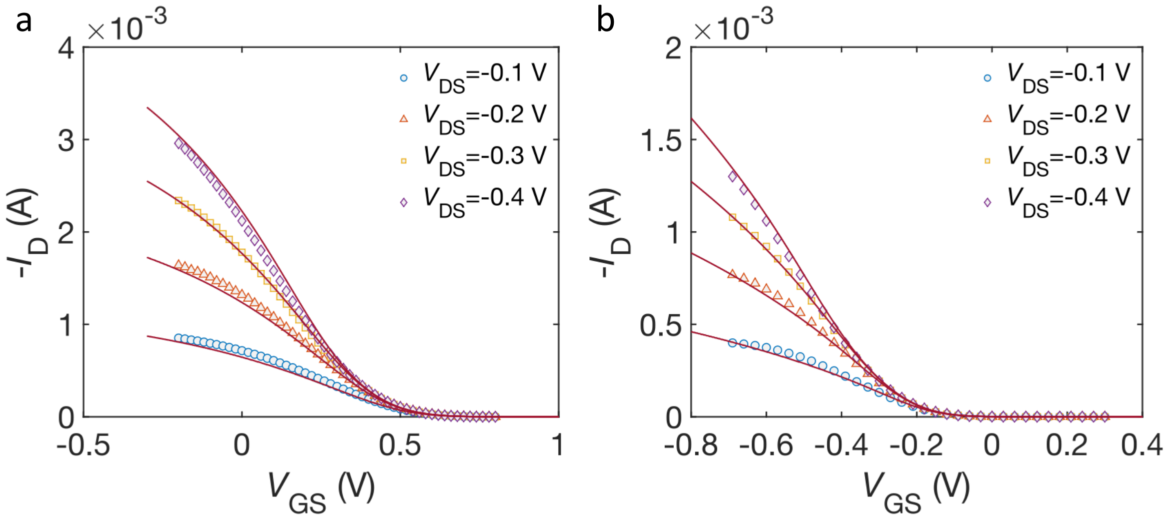

Supplementary Figure 1 | Transfer characteristics of OECTs. Measured (symbols) and modelled (lines) I_D-V_G of OECTs at various drain voltages V_D. **a** PEDOT:PSS OECTs (T_1). **b** p(g2T-TT) OECTs (T_2).

Supplementary Note 1 | OECT drain current model

The OECT drain-current equation is based on the Bernads-Malliaras model including energy disorder as proposed by Friedlein et al.,[21,22] channel length modulation as reported in Romele et al., [23] and ion concentration dependent threshold voltage as demonstrated in Romele et al.,[24] and it reads:

$$I_{\mathrm{D}} = \frac{\Gamma}{\gamma} V_{\mathrm{P}}^{2-\gamma} [\|V_{\mathrm{S}} - V_{\mathrm{G}} + V_{\mathrm{TH}}\|^{\gamma} - \|V_{\mathrm{D}} - V_{\mathrm{G}} + V_{\mathrm{TH}}\|^{\gamma}](1 + \lambda V_{\mathrm{SD}}) \quad (1)$$

where

$$\Gamma = \frac{W}{L} t\mu C_{\mathrm{V}} \quad (2)$$

$$V_{\mathrm{P}} = \frac{q p_0}{\mathrm{C_V}} \quad (3)$$

$$\gamma = \frac{E_0}{k_{\mathrm{B}} T} + 1 \quad (4)$$

$$V_{\mathrm{TH}} = V_{\mathrm{P}} - V_{\mathrm{SH}} + V_{\mathrm{ISM}} \quad (5)$$

where W, L, t, are the channel width, length and thickness, respectively, μ is the hole mobility, C_V is the volumetric capacitance, γ is a parameter accounting for energy disorder, E_0 is the disorder parameter describing the energetic width of the tail of the density of states, λ accounts for channel length modulation, k_{B} is the Boltzmann constant, T is the temperature, V_{TH} is the threshold voltage, q is the elementary charge, p_0 is the intrinsic doping of the semiconductor, V_{SH} accounts for the voltage shift as a function of the ion concentration. In Supplementary Equation (1), we define the $\|\cdot\|$ operator as:

$$\|x\| = x \qquad \text{if } x > 0$$

$$\|x\| = 0 \qquad \text{if } x < 0$$

According to Supplementary Equation (1)-(5), the OECT drain current depends on the applied voltages ($V_{\mathrm{S}}, V_{\mathrm{D}}, V_{\mathrm{G}}$) and on the physical and geometrical parameters ($\Gamma, V_{\mathrm{TH}}, \gamma$). To fit the model with the measurements, we considered the normalized transconductance $g_{\mathrm{m}} = (\partial I_{\mathrm{D}}/\partial V_{\mathrm{SG}})/(V_{\mathrm{S}} - V_{\mathrm{D}})$. In the case of organic mixed ionic-electronic conductors with small energy disorder $\gamma \cong 2$, $g_{\mathrm{m}} = \frac{\Gamma}{\gamma} V_{\mathrm{P}}^{2-\gamma}$, and Supplementary Equation (1) can be re-written as:

$$I_{\mathrm{D}} = g_{\mathrm{m}} [\|V_{\mathrm{S}} - V_{\mathrm{G}} + V_{\mathrm{TH}}\|^{\gamma} - \|V_{\mathrm{D}} - V_{\mathrm{G}} + V_{\mathrm{TH}}\|^{\gamma}] \quad (6)$$

The voltage V_{SH} accounts for the voltage shift as a function of the ion concentration,[24] and it reads:

$$V_{\mathrm{SH}} = V_{\mathrm{GE}} + V_{\mathrm{EP}} \quad (7)$$

$$V_{\mathrm{GE}} = \frac{k_{\mathrm{B}} T}{\mathrm{q}} \log(c) \quad (8)$$

$$V_{\mathrm{EP}} = \frac{k_{\mathrm{B}} T}{\mathrm{q}} \operatorname{asinh}\left(\frac{z_{\mathrm{fix}} N_{\mathrm{fix}}}{2zc}\right) \quad (9)$$

where c is the ion concentration in the electrolyte, z and z_{fix} is the valence of the ions dissolved into the electrolyte and the valence of fixed charge into the polyelectrolyte, respectively, and N_{fix} is the fixed charges in the bulk of the polyelectrolyte. We note that V_{SH} (Supplementary Equation (7)) includes the contributions of the Nernst's potential (V_{GE}, Supplementary Equation (8)) at the non-polarizable gate electrode/electrolyte interface and the Donnan's equilibrium potential (V_{EP}, Supplementary Equation (9)).

When an ion-selective membrane (ISM) is used, the voltage V_{ISM} generated in response to the specific concentration of target ions within the aqueous electrolyte at the analyte/membrane interface can be calculated with the Nernst equation[25–27] and results:

$$V_{\mathrm{ISM}} = V^0 + s_{\mathrm{i}} \ \log_{10}\left\{[M] - \alpha[N]^{\frac{s_{\mathrm{j}}}{s_{\mathrm{i}}}}\right\} \tag{10}$$

where V^0 denotes the formal potential of the electrolyte ion and the work function generated by the connection of materials with different work functions in the overall extended gate architecture, $s_{\mathrm{i}} = \eta_{\mathrm{i}} \frac{k_{\mathrm{B}} T}{z_{\mathrm{i}} e} \ln(10)$, k_{B} is Boltzmann's constant, T is the temperature, z_{i} is the valence of the selected ions, e is the elementary charge, η_{i} is a dimensionless factor between 0 and 1 that accounts for the activity of the selected ions, $[M]$ is the concentration of the selected ions, α is the selectivity coefficient, $s_{\mathrm{j}} = s_{\mathrm{i}} \frac{\eta_{\mathrm{j}} z_{\mathrm{i}}}{\eta_{\mathrm{i}} z_{\mathrm{j}}}$ is the sensitivity of the interfering ions, η_{j} accounts for the activity of the interfering ions, and $[N]$ is the concentration of interfering ions.

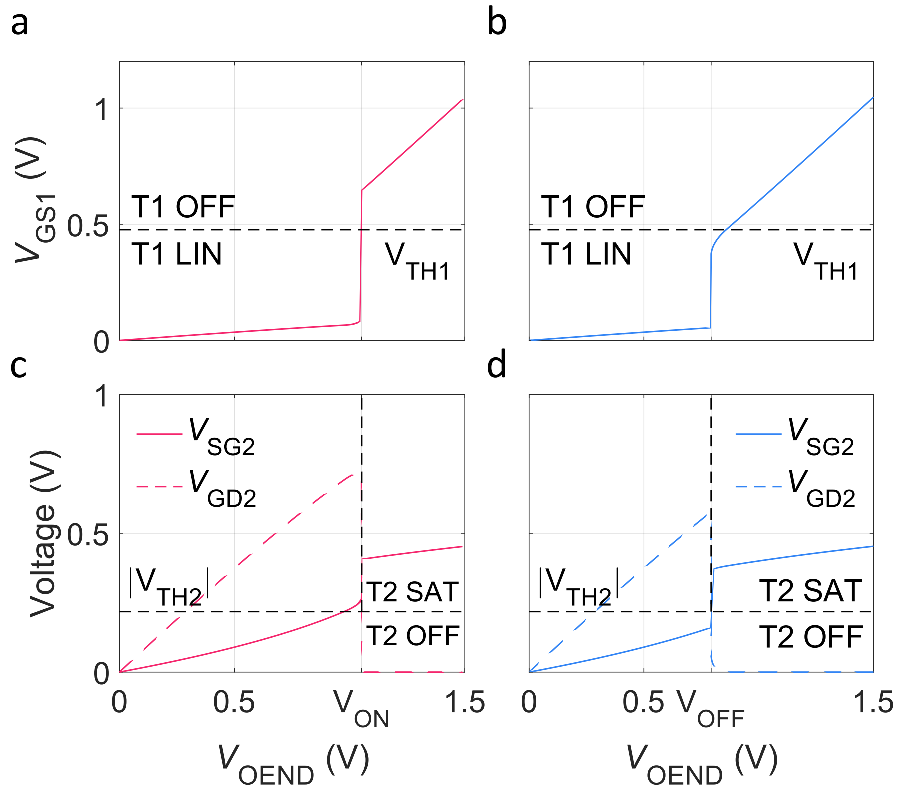

Supplementary Figure 2 | OEND internal voltages. Voltages are calculated by sweeping V_{OEND} from 0 V to 1.5 V (red lines) and back to 0 V (blue lines). **a, b** Gate source voltage of the OECT T_1 as a function of V_{OEND}. The region of operation and the threshold voltage (V_{TH1}) are highlighted. **c, d** Source-gate voltage (V_{SG2}) and gate-drain voltage (V_{GD2}) of the OECT T_2.

Supplementary Note 2 | OEND switching voltages

When the OEND operates in region (1) T_1 is ON and operates in linear region while T_2 is OFF. The transistor T_1 currents reads:

$$I_{\mathrm{D1}} = g_{\mathrm{m1}}[(V_{\mathrm{SG1}} + V_{\mathrm{TH1}})^{\gamma} - (V_{\mathrm{DG1}} + V_{\mathrm{TH1}})^{\gamma}] \tag{11}$$

By increases I_{OEND} ($I_{\mathrm{OEND}} = I_{\mathrm{S1}}$), the voltage-drop on resistor R_1 enhances, the overdrive voltage of T_1 decreases, and when $V_{\mathrm{SD1}} > |V_{\mathrm{T2}}|$ T_2 turns ON. The current and voltage of the OEND immediately before T_1 turns OFF and T_2 turns ON can be calculated as follows:

$$I_{\mathrm{ON}} = g_{\mathrm{m1}}\left[V_{\mathrm{TH1}}^{\gamma_1} - (V_{\mathrm{TH1}} + V_{\mathrm{TH2}})^{\gamma_1}\right] \tag{12}$$

$$V_{\mathrm{ON}} = R_2 I_{\mathrm{ON}} - V_{\mathrm{TH2}} \tag{13}$$

By plugging Supplementary Equation (12) into Supplementary Equation (13), and in the case a low-energy disorder polymer used for the channel T1, e.g. in our case we used PEDOT:PSS, $\gamma_1 \cong 2$ and V_{ON} can be approximated as:

$$V_{\mathrm{ON}} \cong (R_1 + R_2) g_{\mathrm{m1}} \frac{V_{\mathrm{TH2}}(2V_{\mathrm{TH1}} + V_{\mathrm{TH2}})}{2\, g_{\mathrm{m1}}\, R_1\, V_{\mathrm{TH2}} - 1} - V_{\mathrm{TH2}} \tag{14}$$

Then, as T_2 turns ON, the current path R_1-T_2 gives rise to an abrupt increase of the OEND current, T_1 turns OFF as $V_{\mathrm{R1}} > V_{\mathrm{TH1}}$, and the OEND operates in the region (3). Here T_1 is still OFF, making $I_{\mathrm{S1}} = 0\ \mathrm{A}$, $V_{\mathrm{R2}} = 0\ \mathrm{V}$, T_2 is operated saturation and its source-drain current results:

$$I_{\mathrm{S2}} = g_{\mathrm{m2}}(V_{\mathrm{SG2}} + V_{\mathrm{TH2}})^{\gamma_2} \tag{15}$$

$$V_{\mathrm{SG1}} = -V_{\mathrm{TH1}} = V_{\mathrm{R1}} = -I_{\mathrm{S2}} R_1 \rightarrow I_{\mathrm{S2}} = \frac{V_{\mathrm{TH1}}}{R_1} \tag{16}$$

When V_{OEND} decreases, the voltage drop on R_1 decreases as well, and when $V_{\mathrm{R1}} = V_{\mathrm{TH1}}$, T_1 turns ON again (region 4). Combining Supplementary Equation (15) and (16), we obtained:

$$V_{\mathrm{SG2}} = \sqrt[\gamma_2]{\frac{V_{\mathrm{TH1}}}{g_{\mathrm{m2}} R_1}} - V_{\mathrm{TH2}} \tag{17}$$

The OEND circuit topology yields: $V_{\mathrm{OFF}} = V_{\mathrm{SG2}} + V_{\mathrm{R1}}$, that can be re-written as:

$$V_{\mathrm{OFF}} = \sqrt[\gamma_2]{\frac{V_{\mathrm{TH1}}}{\mathrm{g}_{\mathrm{m2}} R_1}} + V_{\mathrm{TH1}} - V_{\mathrm{TH2}} \tag{18}$$

and I_{OFF} reads:

$$I_{\mathrm{OFF}} = \frac{V_{\mathrm{TH1}}}{R_1} \tag{19}$$

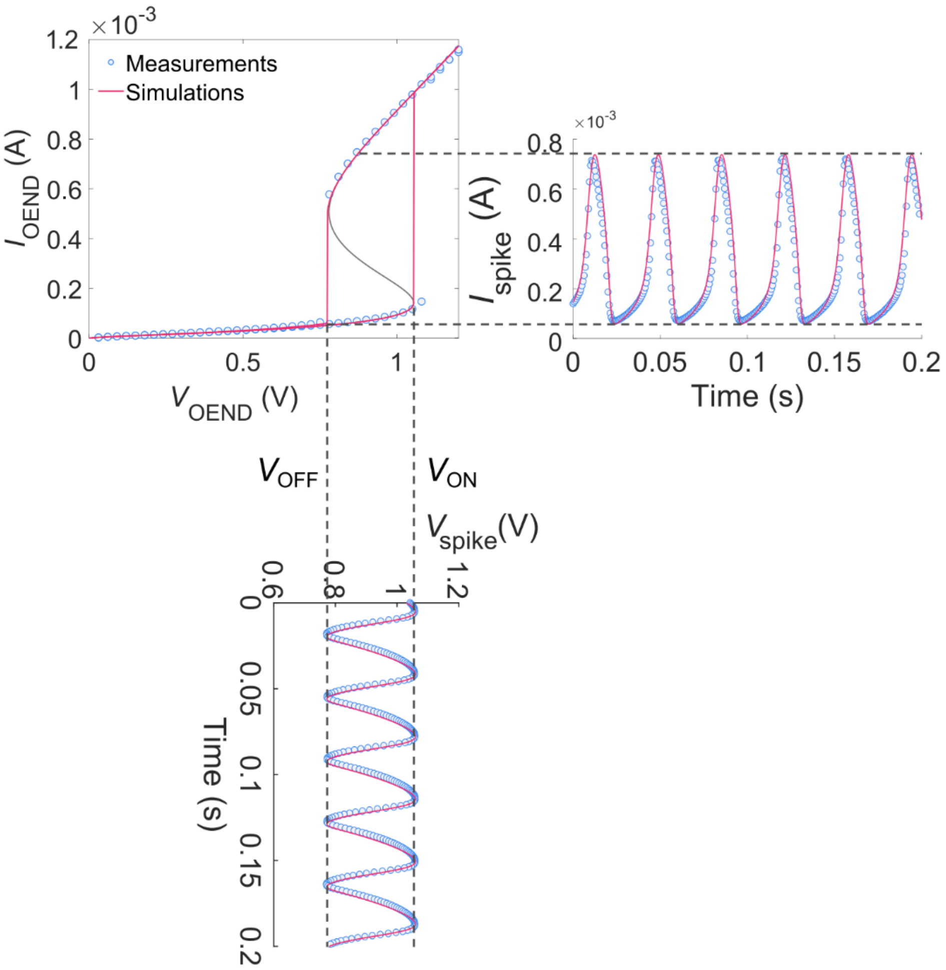

Supplementary Figure 3 | OEND characteristic and OAN spiking amplitude. Relation between non-linear phenomena and electrochemical oscillations. **a** OEND current-voltage characteristic $I_{\mathrm{OEND}}(V_{\mathrm{OEND}})$ accessed in voltage mode (symbols and red line): V_{OEND} is the independent input variable and I_{OEND} is the output. OEND current-voltage characteristic $V_{\mathrm{OEND}}(I_{\mathrm{OEND}})$ accessed in current mode (grey line): I_{OEND} is the independent input variable and V_{OEND} is the output. Symbols are the measurements, red line is calculated with numerical simulations. **b** Measured (symbols) and simulated (full line) spiking voltage (V_{spike}). V_{ON} and V_{OFF} show the voltages as defined in Supplementary Note 3. **c** Measured (symbols) and simulated (full line) spiking current (I_{spike}).

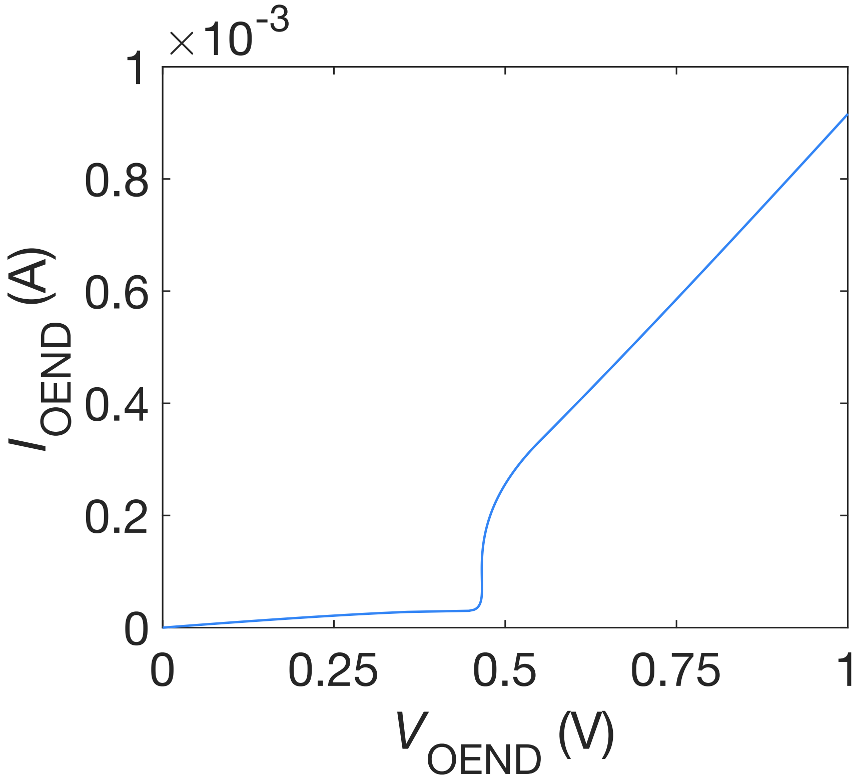

Supplementary Figure 4 | OEND limit condition. Electrical characteristic of the OEND when $V_{\mathrm{TH1}} = |V_{\mathrm{TH2}}|$. In the limit condition the electrical characteristics $I_{\mathrm{OEND}}(V_{\mathrm{OEND}})$ and $V_{\mathrm{OEND}}(I_{\mathrm{OEND}})$ overlap.

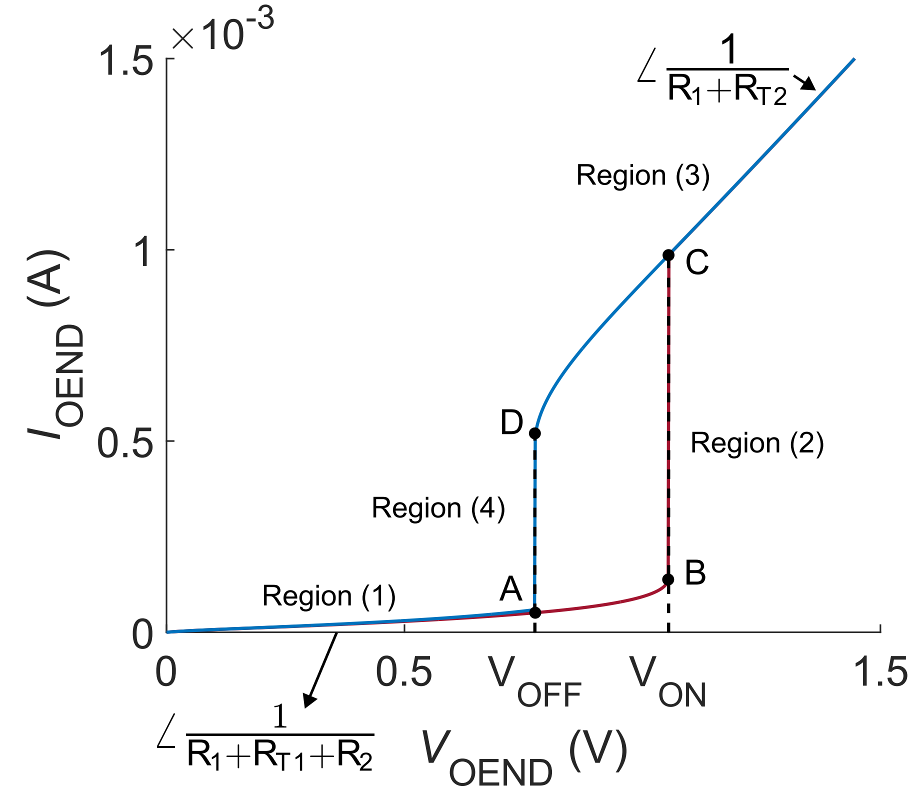

Supplementary Figure 5 | OEND characteristic. Electrical characteristic of the OEND when operating in voltage mode, with notes giving further details of the curve: the vertex of the hysteresis with the capital letters, the regions of the curves and the steepness of Region (1) and (3).

Supplementary Note 3 | Spiking frequency

When the OAN is spiking, viz. above the excitation threshold, capacitor C_L is charged and discharged. Its charging and discharging currents depend on the OEND and load-line parameters. The voltage across C_L corresponds to the spiking voltage of the OAN (V_{spike}), and the displacement current flowing through C_L I_{C} can be calculated as follows:

$$I_{\mathrm{C}} = \frac{V_{\mathrm{IN}} - V_{\mathrm{spike}}}{R_{\mathrm{L}}} - I_{\mathrm{spike}} = \frac{V_{\mathrm{IN}} - V_{\mathrm{spike}} - R_{\mathrm{L}} I_{\mathrm{spike}}}{R_{\mathrm{L}}} \quad (20)$$

where $I_{\mathrm{spike}} = I_{\mathrm{OEND}}$. By substituting Supplementary Equation (20) in the differential capacitor equation $I_C = C_{\mathrm{L}} \frac{\mathrm{d}V_{\mathrm{spike}}}{\mathrm{d}t}$, results that:

$$\mathrm{d}t = \frac{R_{\mathrm{L}} C_{\mathrm{L}}}{V_{\mathrm{IN}} - V_{\mathrm{spike}} - R_{\mathrm{L}} I_{\mathrm{spike}}} \mathrm{d}V_{\mathrm{spike}} \quad (21)$$

When the OAN is spiking, the spiking oscillations V_{spike}-I_{spike} follow the trajectory A-B-C-D highlighted in the Supplementary Figure 5.

Then, integrating Supplementary Equation (21) between the points A-B and the points C-D, we obtain:

$$f_{\mathrm{spike}} = \frac{1}{R_{\mathrm{L}} C_{\mathrm{L}}} \left[\int_{V_{\mathrm{OFF}}(\mathrm{A})}^{V_{\mathrm{ON}}(\mathrm{B})} \frac{\mathrm{d}V_{\mathrm{spike}}}{V_{\mathrm{IN}} - V_{\mathrm{spike}} - R_{\mathrm{L}} I_{\mathrm{spike}}(V_{\mathrm{spike}})} + \int_{V_{\mathrm{ON}}(\mathrm{C})}^{V_{\mathrm{OFF}}(\mathrm{D})} \frac{\mathrm{d}V_{\mathrm{spike}}}{V_{\mathrm{IN}} - V_{\mathrm{spike}} - R_{\mathrm{L}} I_{\mathrm{spike}}(V_{\mathrm{spike}})} \right]^{-1} \quad (22)$$

We note that in the range A-B and C-D the OEND operates in region (1) and region (3), respectively (see the figure above), and I_{spike} can be approximated as:

$$I_{\mathrm{spike}}(V_{\mathrm{spike}})\big|_{\mathrm{A-B}} = \frac{V_{\mathrm{spike}}}{R_1 + R_2 + R_{\mathrm{T1}}} \quad (23)$$

$$I_{\mathrm{spike}}(V_{\mathrm{spike}})\big|_{\mathrm{C-D}} = \frac{V_{\mathrm{spike}} + V_{\mathrm{TH2}}}{R_1 + R_{\mathrm{T2}}} \quad (24)$$

where R_{T1} and R_{T2} are the channel resistance of transistor T_1 and T_2, respectively.

The simulations show that R_{T1} is negligible in Supplementary Equation (23) while in Supplementary Equation (24) R_{T2} is comparable to R_1.

In region (3) T_1 is OFF, $V_{\mathrm{DG2}} = 0\ \mathrm{V}$, $V_{\mathrm{SD2}} = V_{\mathrm{SG2}}$, $V_{\mathrm{D2}} - V_{\mathrm{G2}} + V_{\mathrm{TH2}} < 0\ \mathrm{V}$, and hence $V_{\mathrm{SG2}} = \sqrt[\gamma_2]{\frac{I_{\mathrm{spike}}}{g_{\mathrm{m2}}}} - \mathrm{V_{TH2}}$. $\mathrm{R_{T2}}$ is calculated as $\partial V_{\mathrm{SG2}} / \partial I_{\mathrm{OEND}}$, and reads:

$$R_{\mathrm{T2}} = \sqrt{\frac{g_{\mathrm{m2}}}{I_{\mathrm{spike}}}} \quad (25)$$

By plugging Supplementary Equations (23)-(25) in Supplementary Equation (22), the analytic expression of f_{spike} results:

$$f_{\mathrm{spike}} = \frac{1}{R_{\mathrm{L}} C_{\mathrm{L}}} \left[\frac{R_1 + R_2}{R_1 + R_2 + R_{\mathrm{L}}} \log\left(\frac{(R_1 + R_2) V_{\mathrm{IN}} - (R_1 + R_2 + R_{\mathrm{L}}) V_{\mathrm{OFF}}}{(R_1 + R_2) V_{\mathrm{IN}} - (R_1 + R_2 + R_{\mathrm{L}}) V_{\mathrm{ON}}} \right) + \right.$$

$$\left. \frac{R_1 + R_{\mathrm{T2}}}{R_1 + R_{\mathrm{T2}} + R_{\mathrm{L}}} \log\left(\frac{(R_1 + R_{\mathrm{T2}})(V_{\mathrm{IN}} - R_{\mathrm{L}} I_0) - (R_1 + R_{\mathrm{T2}} + R_{\mathrm{L}}) V_{\mathrm{ON}}}{(R_1 + R_{\mathrm{T2}})(V_{\mathrm{IN}} - R_{\mathrm{L}} I_0) - (R_1 + R_{\mathrm{T2}} + R_{\mathrm{L}}) V_{\mathrm{OFF}}} \right) \right]^{-1} \quad (26)$$

where $I_0 = V_{\mathrm{TH2}} / (R_1 + R_{\mathrm{T2}})$.

Supplementary Equation (26) shows that f_{spike} non-linearly dependents on the OECT geometrical and material parameters embedded in the expressions of V_{ON} and V_{OFF}, see

Supplementary Equations (13)-(18). The Supplementary Equations (1)-(7) also gives the relationship between the threshold of these two transistors and the ionic concentration inside the electrolyte.

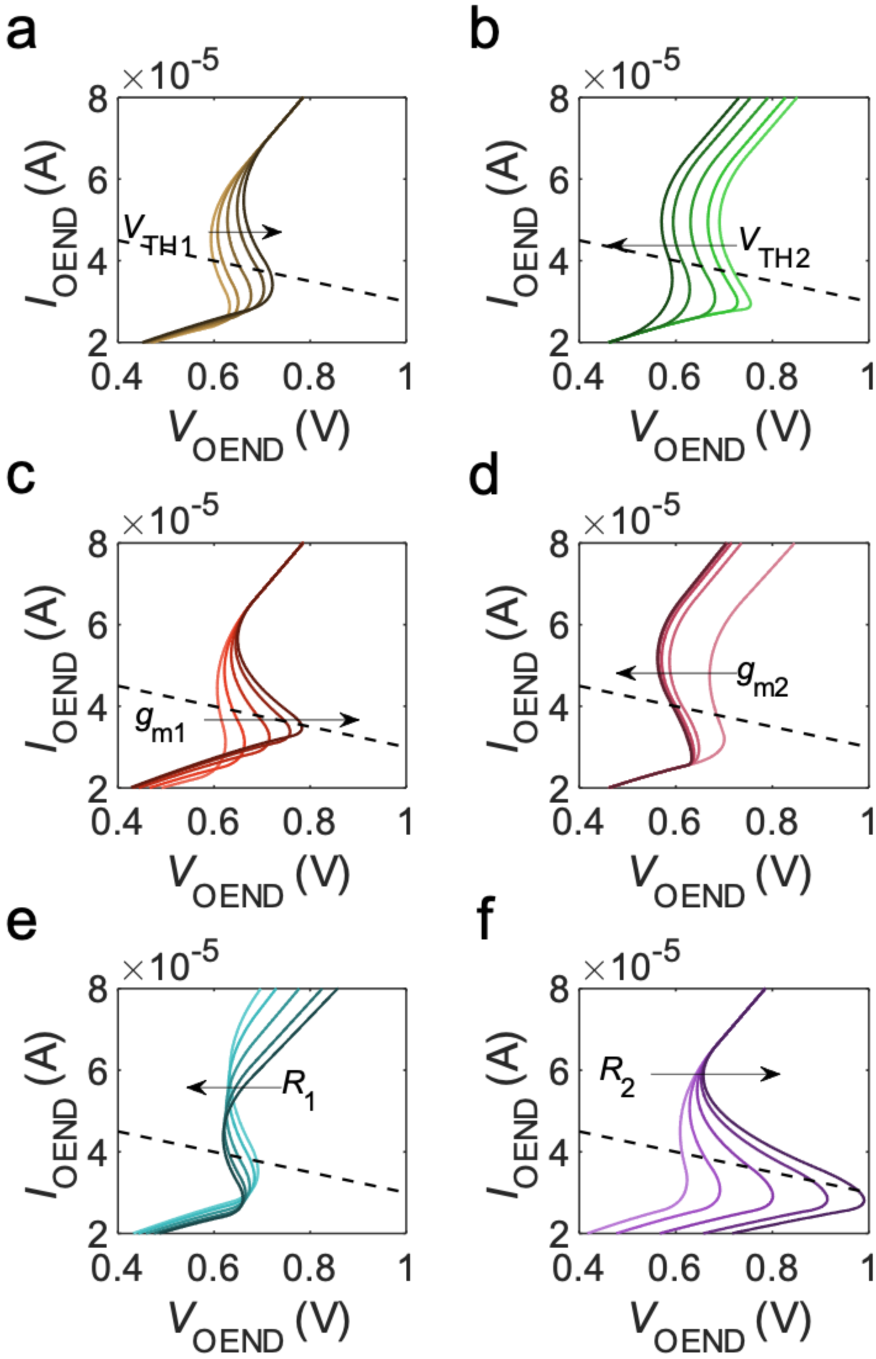

Supplementary Figure 6 | OEND characteristics as a function of device parameters. The OEND is accessed in current mode, $V_{\mathrm{OEND}}(I_{\mathrm{OEND}})$. **a** Threshold voltage of transistor T_1, V_{TH1}, ranges from 0.41 V to 0.47 V. **b** Threshold voltage of transistor T_2, V_{TH2}, ranges from −0.21 V to -0.09 V. **c** Transconductance normalized to the source-drain voltage of transistor T_1, g_{m1}, ranges from 8 10^{-6} S V^{-1} to 17 10^{-6} S V^{-1}. **d** Transconductance normalized to the source-drain voltage of transistor T_2, g_{m2}, ranges from 6 10^{-6} S V^{-1} to 2.42 10^{-4} S V^{-1}. **e** Resistance of resistor R_1, ranges from 5.5 kΩ to 7.5 kΩ. **f** Resistance of resistor R_2, ranges from 10 kΩ to 25 kΩ. The dashed lines in all panels represent the load-line, aiding in showing changes of the crossing point as the parameter varies, while the arrows indicate the direction of parameter increase.

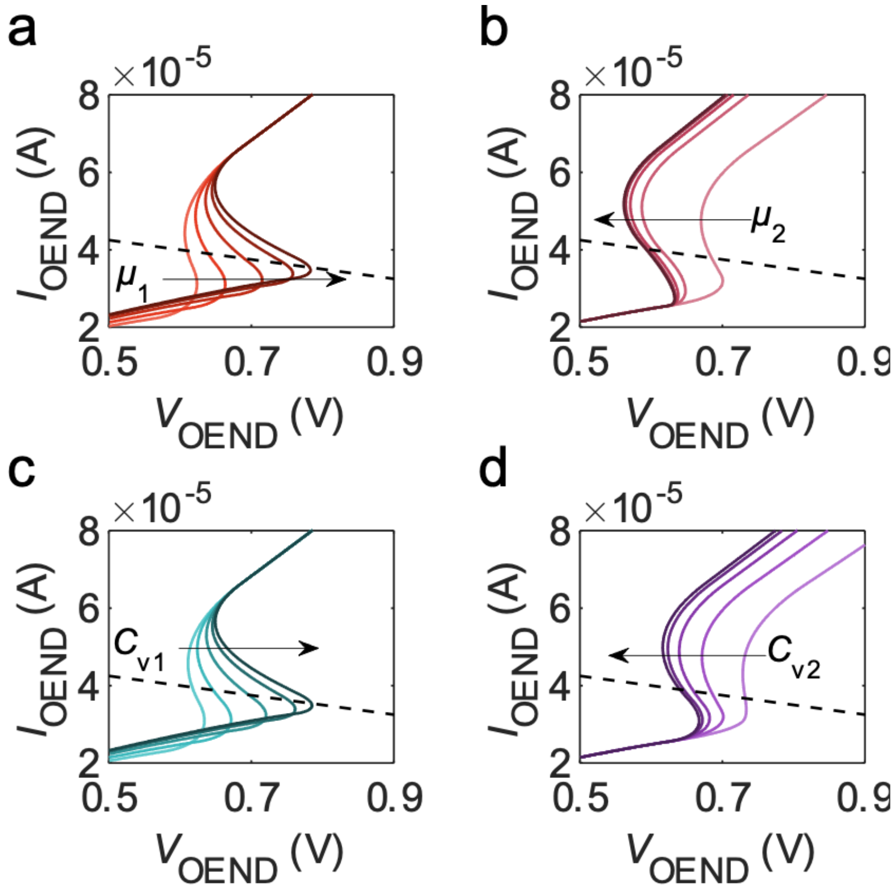

Supplementary Figure 7 | OEND characteristics as a function of material parameters. The OEND is accessed in current mode, $V_{\mathrm{OEND}}(I_{\mathrm{OEND}})$. **a** OMIEC volumetric capacitance of transistor T_1, C_{V1}, ranges from 33 F cm^{-3} to 67 F cm^{-3}. **b** OMIEC volumetric capacitance of transistor T_2, C_{V2}, ranges from 40 F cm^{-3} up to 300 F cm^{-3}. **c** OMIEC electronic mobility of T_1, μ_1, ranges from 0.8 cm^2 V^{-1} s^{-1} to 1.7 cm^2 V^{-1} s^{-1}. **d** OMIEC electronic mobility of T_2, μ_2, ranges from 0.05 cm^2 V^{-1} s^{-1} to 2 cm^2 V^{-1} s^{-1}. The dashed lines in all panels represent the load-line, aiding in showing changes of the crossing point as the parameter varies, while the arrows indicate the direction of parameter increase.

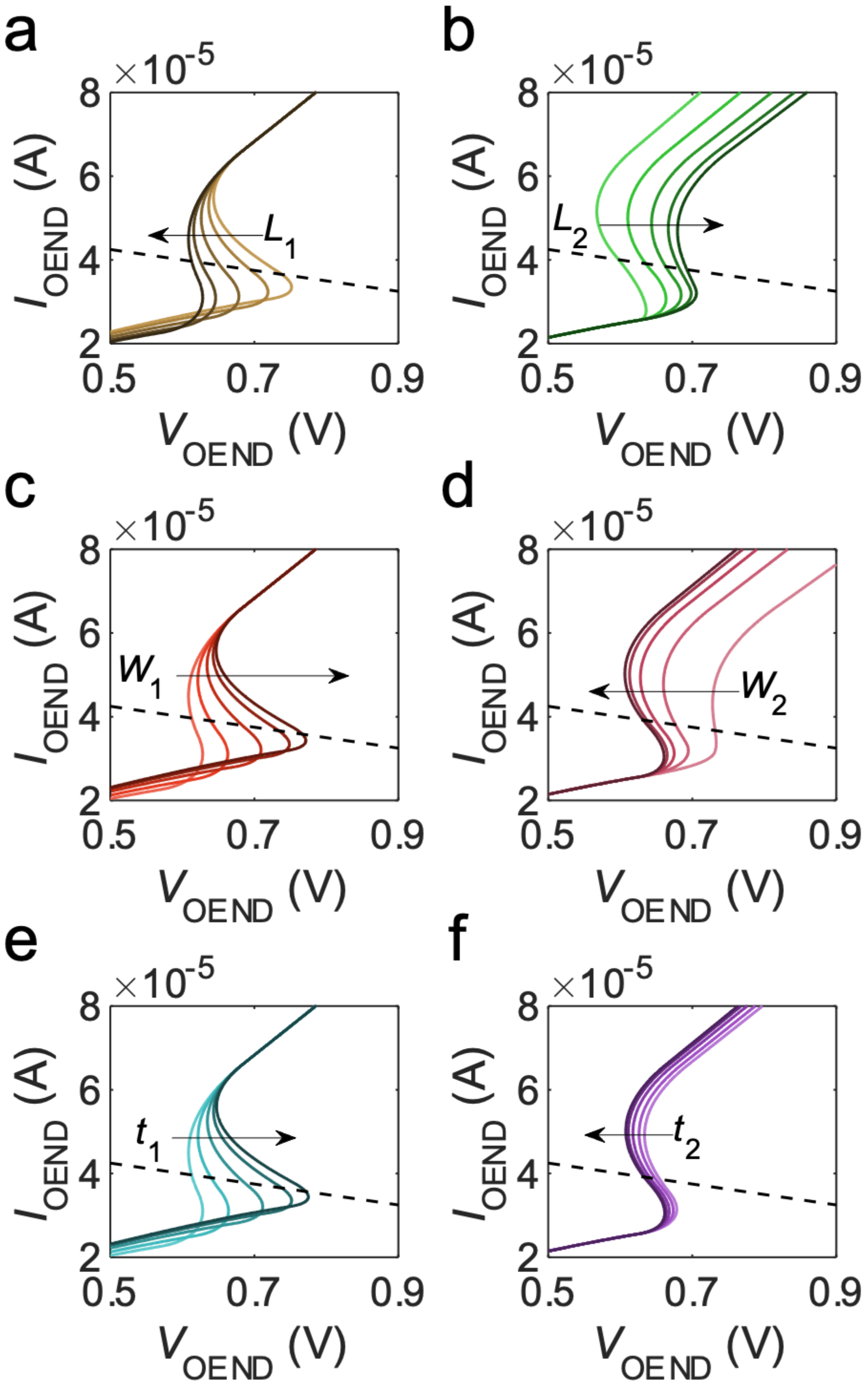

Supplementary Figure 8 | OEND characteristics as a function of geometrical parameters. The OEND is accessed in current mode, $V_{OEND}(I_{OEND})$. **a** Channel width transistor T_1, W_1, ranges from 32 μm to 63 μm. **b** Channel width transistor T_2, W_2, ranges from 50 μm to 500 μm. **c** Channel length transistor T_1, L_1, ranges from 25 μm to 45 μm. **d** Channel length transistor T_2, L_2, ranges from 1 μm to 30 μm. **e** Channel thickness of transistor T_1, t_1, ranges from 80 nm to 160 nm. **f** Channel thickness of transistor T_2, t_2, ranges from 80 nm to 160 nm. The dashed lines in all panels represent the load-line, aiding in showing changes of the crossing point as the parameter varies, while the arrows indicate the direction of parameter increase.

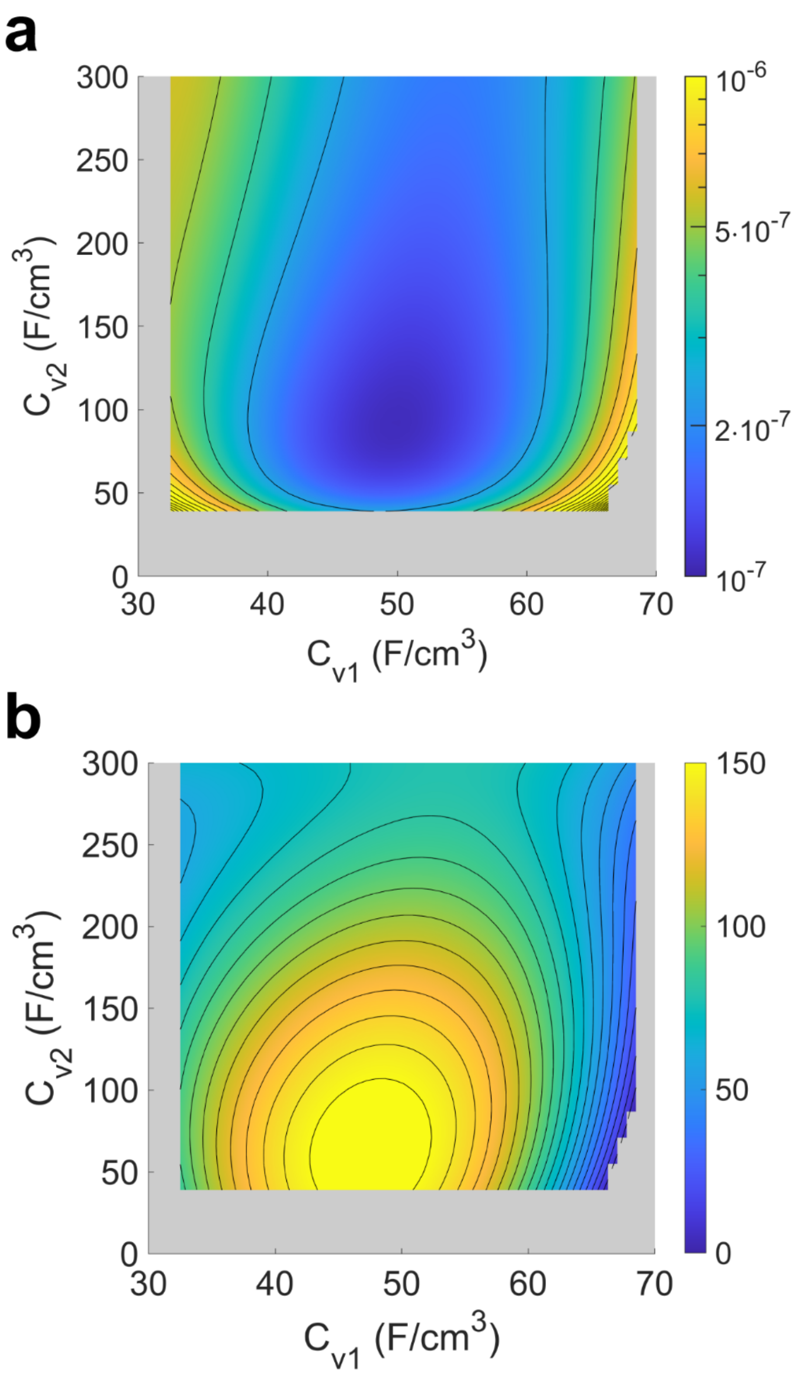

Supplementary Figure 9 | OAN spiking frequency analysis. Investigation of the spiking frequency, f_{spike}, as a function of the volumetric capacitances C_{V1} and C_{V2} of the OECT T_1 and T_2, respectively. **a** Minimum load capacitor, C_{L}, required for OAN spiking for each combination of C_{V1} and C_{V2}. The colorbar represents such values of C_{L}. **b** f_{spike} as a function of C_{V1} and C_{V2} with minimum C_{L}. The gray regions indicate cases of non-spiking OAN. The colorbar represents such values of f_{spike}.

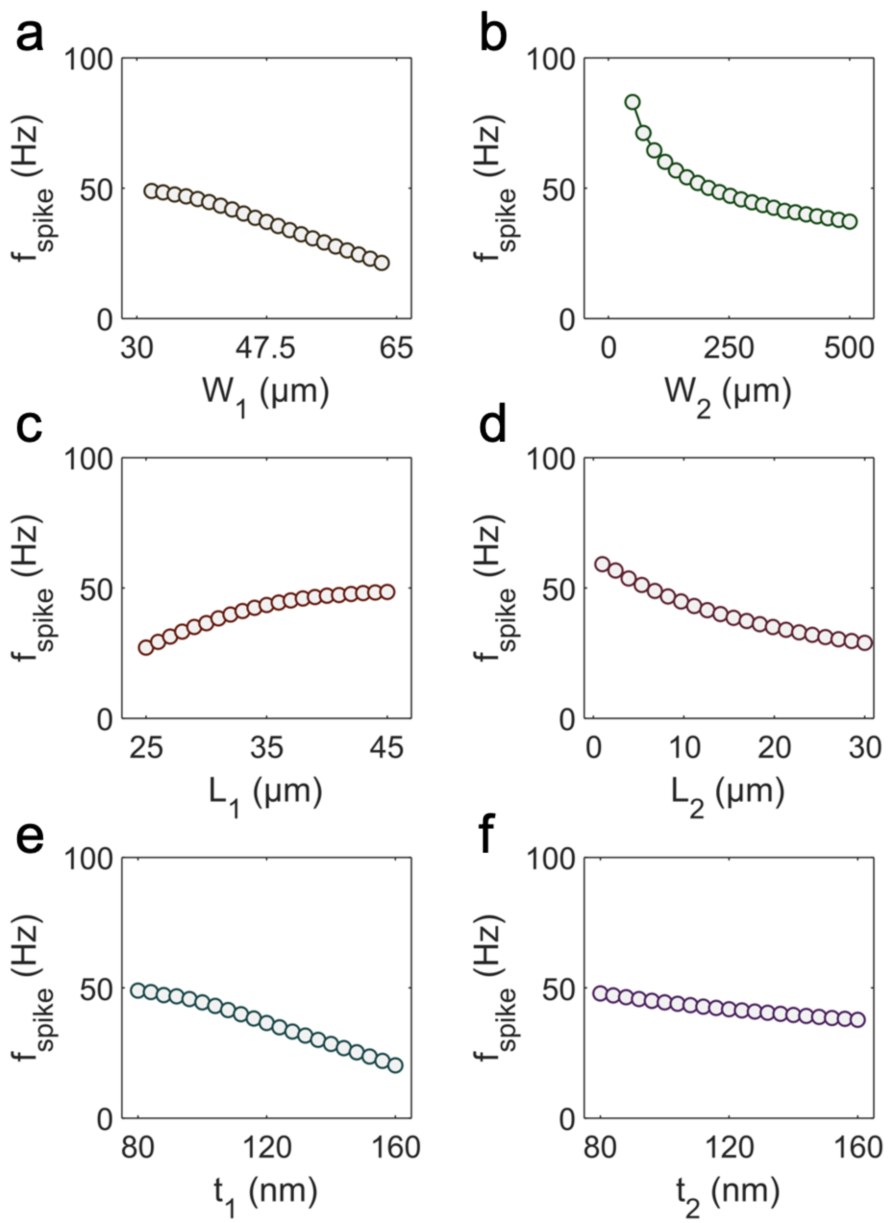

Supplementary Figure 10 | OAN spiking frequency as a function of geometrical parameters. Investigation of the spiking frequency, f_{spike}, as a function of the OMIEC geometrical parameters: **a** channel width of transistor T_1, W_1, **b** channel width of transistor T_2, W_2, **c** channel length of transistor T_1, L_1, **d** channel length of transistor T_2, L_2, **e** channel thickness of transistor T_1, t_1, and **f** channel of transistor T_2, t_2. It is observed that reducing the dimensions such as W_1, W_2, L_2, t_1, and $t_2$leads to an increase in spiking frequency (f_{spike}), particularly notable in the case of W_2. Conversely, f_{spike}decreases with an increase in L_1. This pattern is attributed to the corresponding reduction in OECT capacitance with decreasing geometries. The opposite trend with $f_{\mathrm{spike}}$is explained by the decrease in T_1 conductivity (and consequently drain current) with increasing f_{spike}, resulting in a lower V_{ON}, as evidenced by the amplitude of the spikes in Supplementary Fig. 12 (panel c).

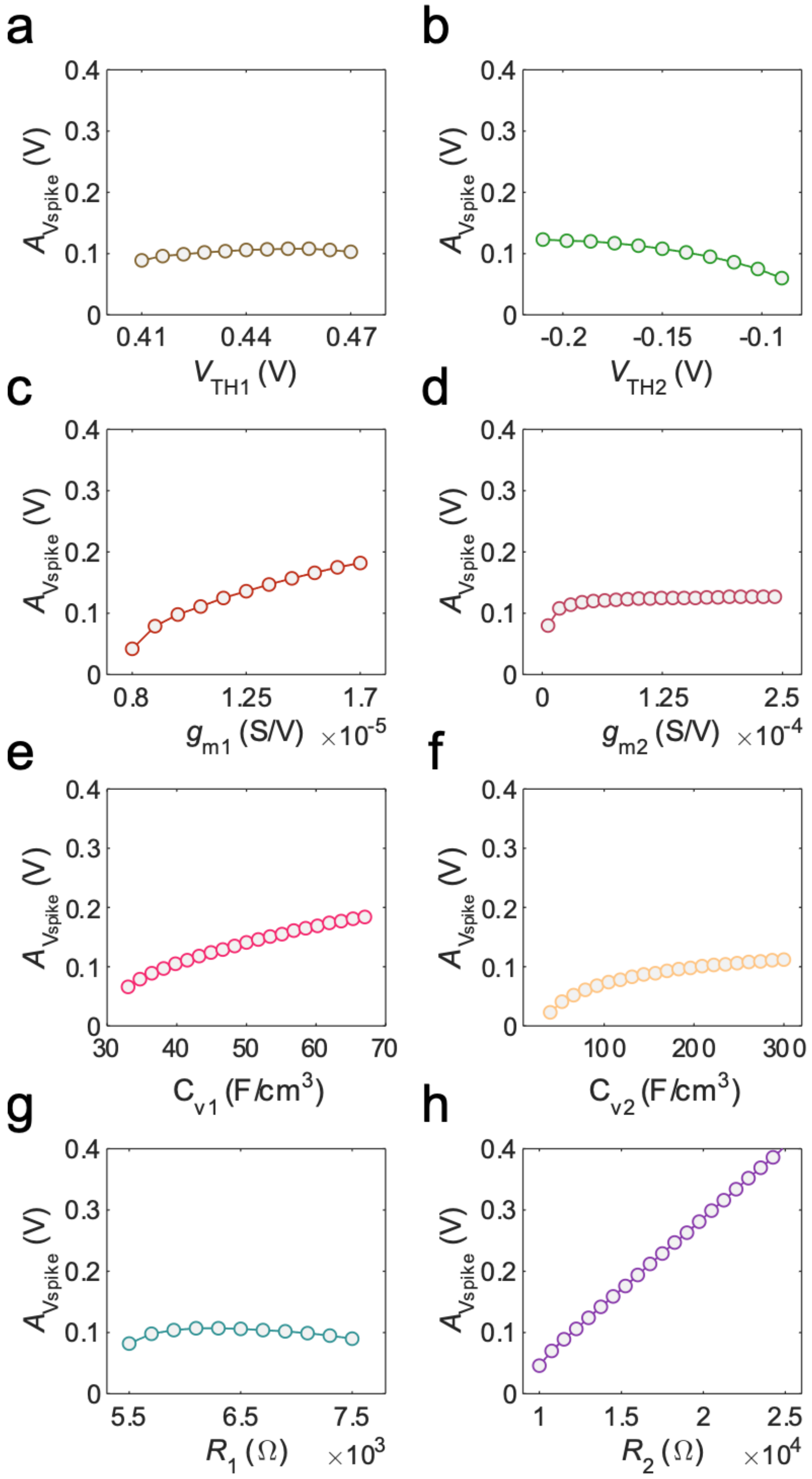

Supplementary Figure 11 | Amplitude of OAN spiking voltage vs materials and device parameters. Investigation of the voltage spiking amplitude A_{Vspike} as a function of OAN materials and device parameters: **a** threshold voltage of T_1, V_{TH1}, **b** threshold voltage of T_2, V_{TH2}, **c** normalized transconductance of T_1, g_{m1}, **d** normalized transconductance of T_2, g_{m2}, **e** volumetric capacitance of T_1, C_{v1}, **f** volumetric capacitance of T_2, C_{v2}, **g** resistance of resistor R_1, and **h** resistance of resistor R_2.

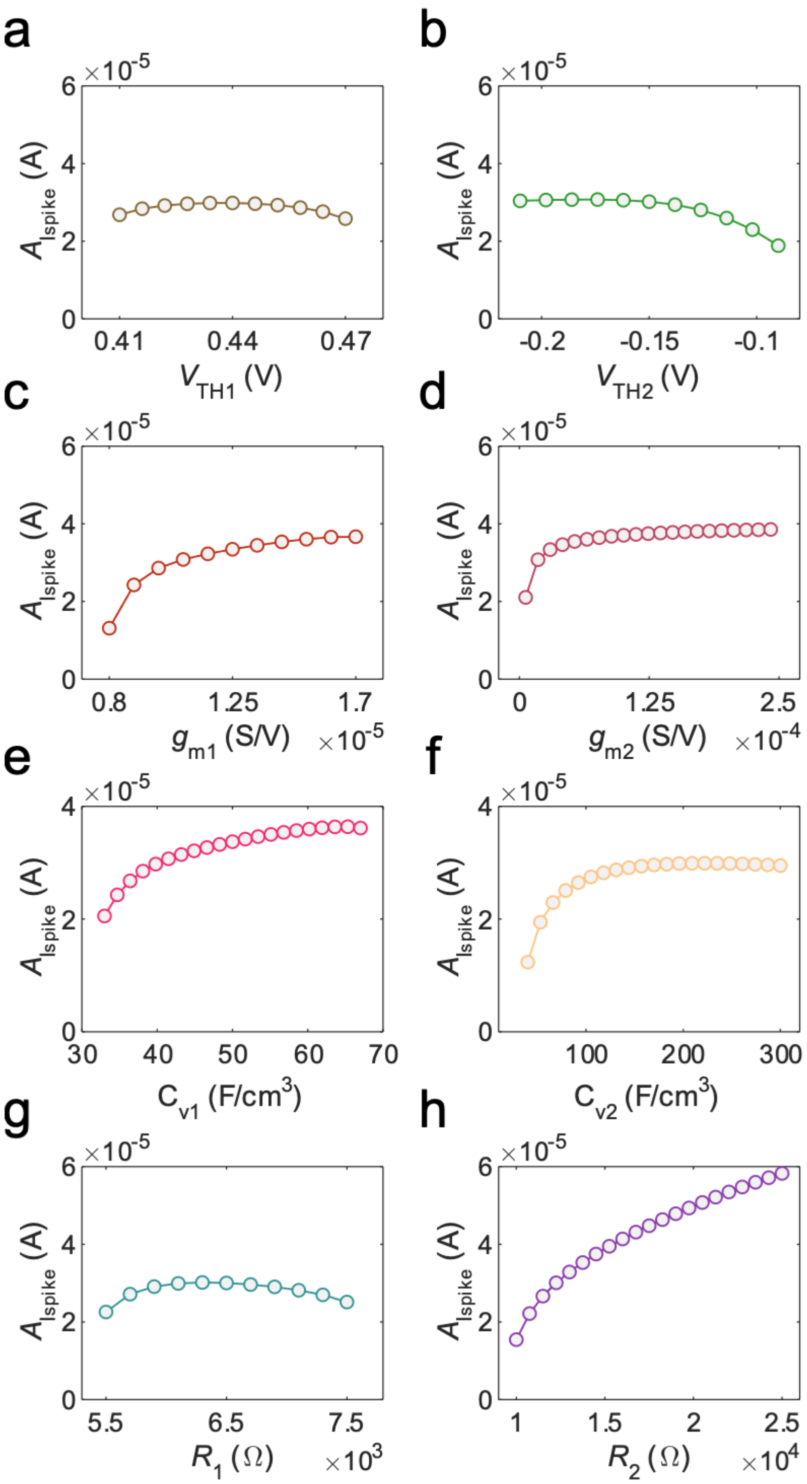

Supplementary Figure 12 | Amplitude of OAN spiking current vs materials and device parameters. Investigation of the current spiking amplitude A_{Ispike} as a function of OAN materials and device parameters: **a** threshold voltage of T_1 V_{TH1}, **b** threshold voltage of T_2 V_{TH2}, **c** normalized transconductance of T_1 g_{m1}, **d** normalized transconductance of T_2 g_{m2}, **e** volumetric capacitance of T_1 C_{v1}, **f** volumetric capacitance of T_2 C_{v2}, **g** resistance of resistor R_1, and **h** resistance of resistor R_2.

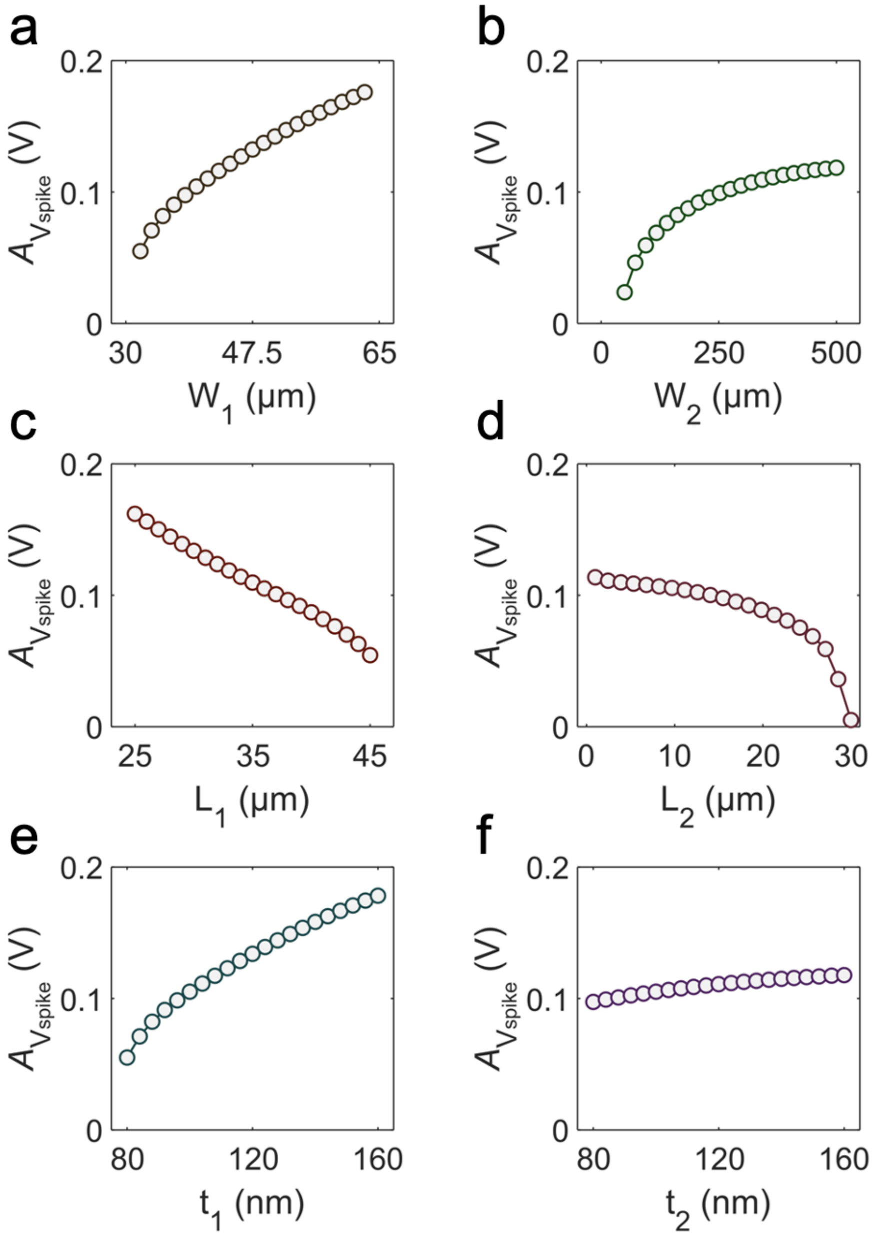

Supplementary Figure 13 | Amplitude of OAN spiking voltage vs geometrical parameters. Investigation of the voltage spiking amplitude A_{Vspike} as a function of OMIEC geometrical parameters: **a** channel width of transistor T_1, W_1, **b** channel width of transistor T_2, W_2, **c** channel length of transistor T_1, L_1, **d** channel length of transistor T_2, L_2, **e** channel thickness of transistor T_1, t_1, and **f** channel of transistor T_2, t_2.

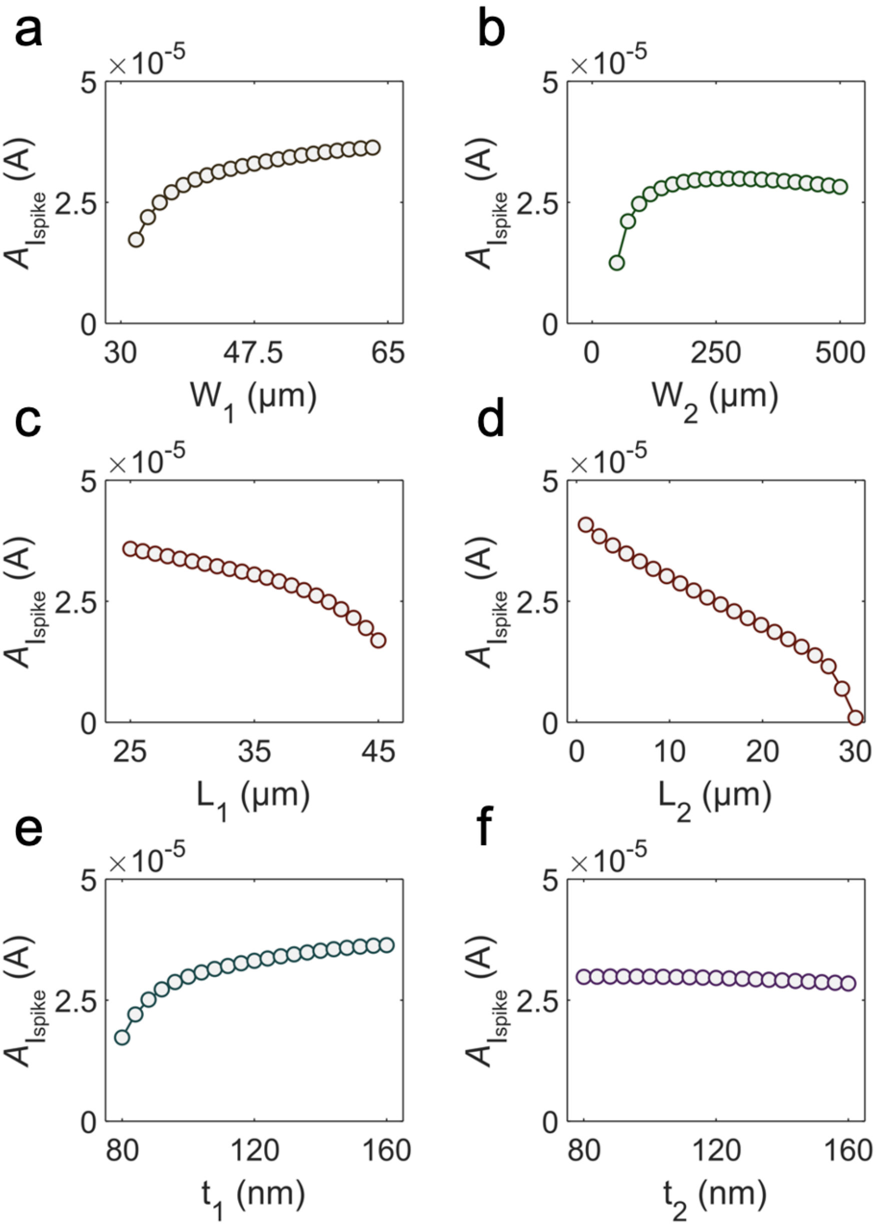

Supplementary Figure 14 | Amplitude of OAN spiking current vs geometrical parameters. Investigation of the current spiking amplitude A_{Ispike} as a function of OMIEC geometrical parameters: **a** channel width of transistor T_1, W_1, **b** channel width of transistor T_2, W_2, **c** channel length of transistor T_1, L_1, **d** channel length of transistor T_2, L_2, **e** channel thickness of transistor T_1, t_1, and **f** channel of transistor T_2, t_2.

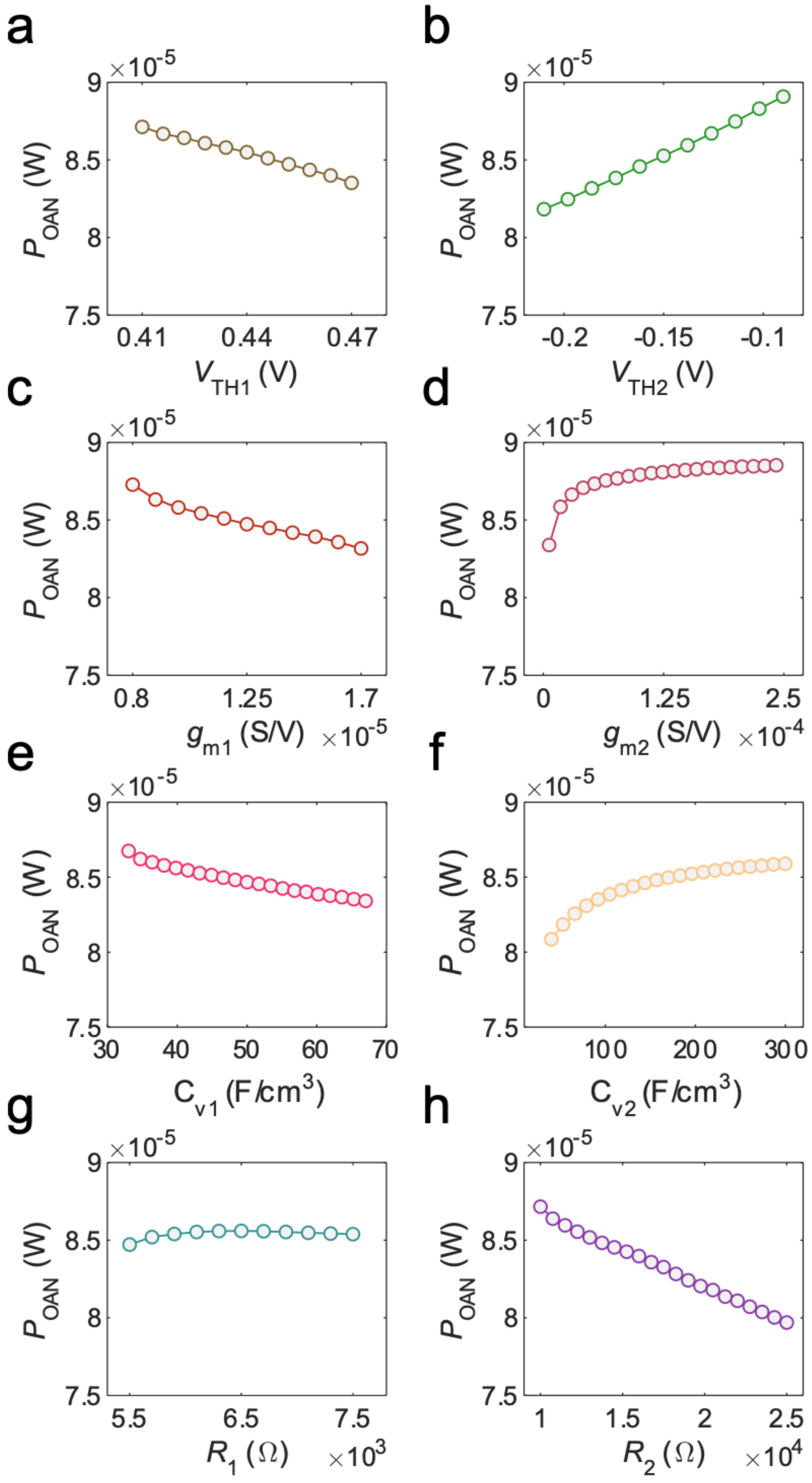

Supplementary Figure 15 | OAN power consumption vs material and device parameters. Investigation of the OAN power consumption P_{OAN} as a function of **a** threshold voltage of T_1, V_{TH1}, **b** threshold voltage of T_2, V_{TH2}, **c** normalized transconductance of T_1, g_{m1}, **d** normalized transconductance of T_2, g_{m2}, **e** volumetric capacitance of T_1, C_{V1}, **f** volumetric capacitance of T_2, C_{V2}, **g** resistance of resistor R_1, and **h** resistance of resistor R_2. P_{OAN} reduces with an increase

in the threshold voltage (V_{TH1}, panel a) and the normalized transconductance (g_{m1}, panel c) of the OECT T_1. This is because a lower voltage drop occurs across the channel of T1 when it operates in the linear region. Similarly, increasing R_2 (panel f) reduces the current flowing in the branch R_1-R_{T1} and R_2, consequently lowering the power consumption. Conversely, P_{OAN} increases with an increase in V_{TH2} (panel b) since T_2 turns ON at lower voltages. We observed that for P_{OAN} increases with g_{m2} for small values of g_{m2} and then a plateau is obtained (panel d). This is because the current flowing in the branch R_1-T_2 is limited by R_1 when T_2 is ON and increasing g_{m2} results that $R_{\mathrm{T2}} < R_1$. While R_1 limits the current in the R_1-T_2 branch, the voltage across R_1 contributes to both turning OFF T_1 (reducing P_{OAN}) and turning ON T_2 (increasing P_{OAN}), explaining the slight increase in P_{OAN} with R_1 (panel e).

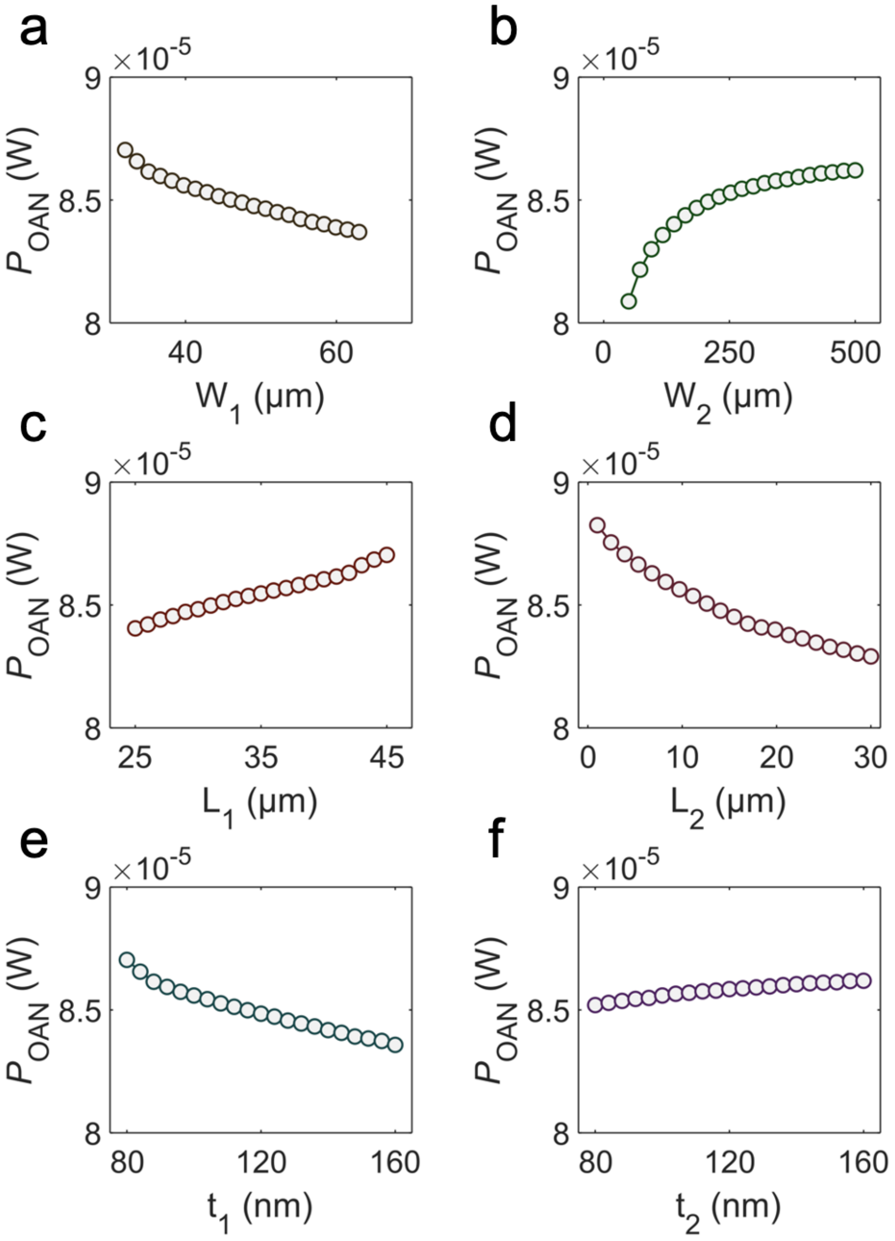

Supplementary Figure 16 | OAN power vs geometrical parameters. Investigation of the OAN power consumption P_{OAN} as a function of as a function of OMIEC geometrical parameters: **a** channel width of transistor T_1, W_1, **b** channel width of transistor T_2, W_2, **c** channel length of transistor T_1, L_1, **d** channel length of transistor T_2, L_2, **e** channel thickness of transistor T_1, t_1, and **f** channel of transistor T_2, t_2. P_{OAN} diminishes with increased W_1, L_2, and t_1, whereas it decreases in other cases. Notably, reducing W_2 emerges as the most effective means to minimize power consumption. By reducing W_2, T_2 conductivity decreases, thereby minimizing current in the R_1-T_2 branch of the OEND.

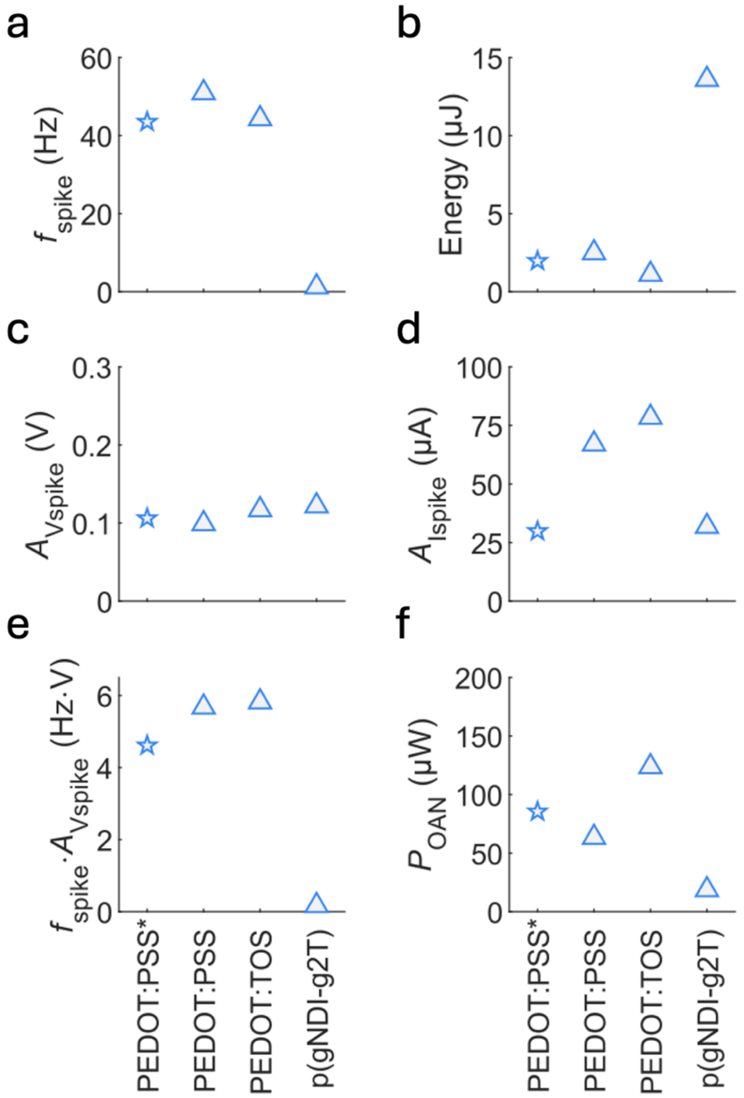

Supplementary Figure 17 | OAN performance with various OMIEC materials for depletion-mode OECTs (T_1). Investigation of the OAN performance considering various OMIEC materials suitable for the fabrication of depletion-mode transistor T_1. The parameters considered are: **a** spiking frequency f_{spike}, **b** spiking energy, **c** spiking voltage amplitude A_{Vspike}, **d** spiking current amplitude A_{Ispike}, **e** spiking frequency multiplied by the spiking voltage amplitude and **f** the power consumption P_{OAN}. The fabricated OAN is based on the material PEDOT:PSS for transistor T_1 and p(g2T-TT) for transistor T_2. The star symbols indicate the materials and the corresponding parameters used in our work. The parameters of our materials are the following. PEDOT:PSS*: volumetric capacitance C_{V} = 43 F cm^{-3}, hole mobility μ = 1.02 cm^{2} V^{-1} s^{-1}, threshold voltage V_{TH} = 0.477 V. p(g2T-TT)*: C_{V} = 241 F cm^{-3}, μ = 0.13 cm^{2} V^{-1} s^{-1}, and V_{TH} = -0.23 V. The performance estimated with the state-of-art materials are calculated by substituting the material parameters of transistor T_1 with those of the material under investigation. The parameters of the materials investigated (triangle symbols) are taken from Refs.[28–32], and are the following. PEDOT:PSS: C_{V}= 39 F cm^{-3}, μ = 1.9 cm^{2} V^{-1} s^{-1}, V_{TH} = 0.4 V. PEDOT:TOS: C_{V}= 136 F cm^{-3}, μ = 0.93 cm^{2} V^{-1} s^{-1}, and V_{TH} = 0.52 V. p(gNDI-g2T): C_{V} = 397 F cm^{-3}, μ = 1 10^{-4} cm^{2} V^{-1} s^{-1}, and V_{TH} = 0.35 V.

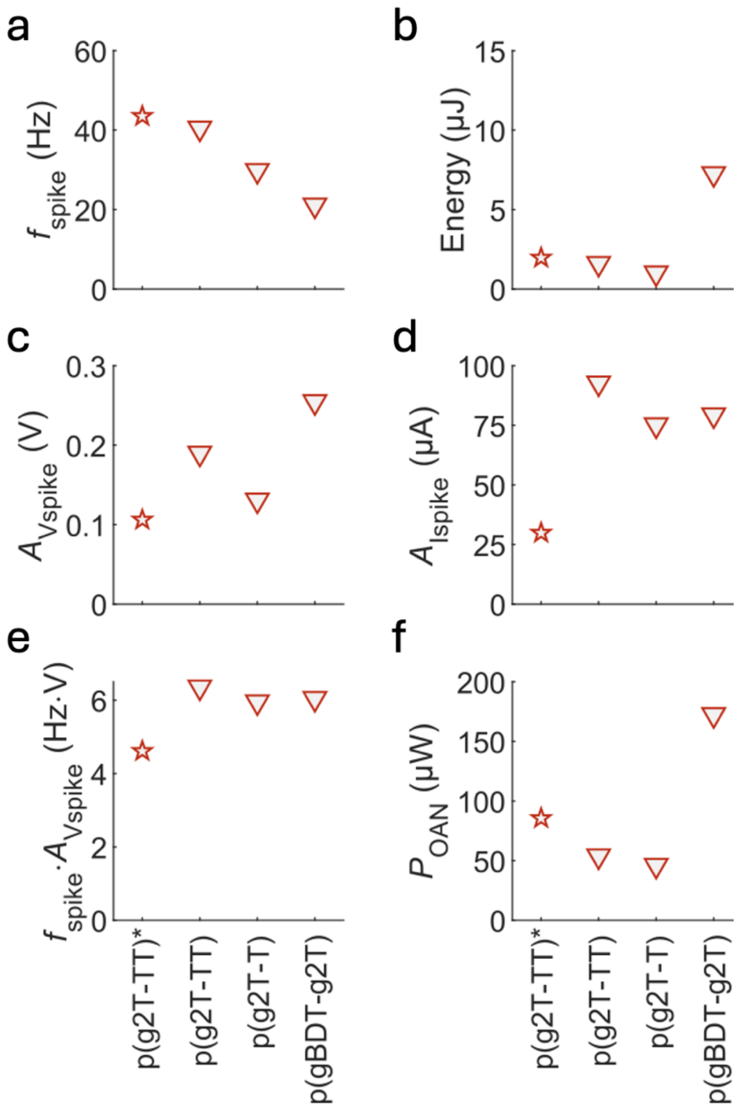

Supplementary Figure 18 | OAN performance with various OMIEC materials for accumulation mode OECTs (T_2). Investigation of the OAN performance considering various OMIEC materials suitable for the fabrication of accumulation-mode transistor T_2. The parameters considered are: **a** spiking frequency f_{spike}, **b** spiking energy, **c** spiking voltage amplitude A_{Vspike}, **d** spiking current amplitude A_{Ispike}, **e** spiking frequency multiplied by the spiking voltage amplitude and **f** the power consumption P_{OAN}. The fabricated OAN is based on the material PEDOT:PSS for transistor T_1 and p(g2T-TT) for transistor T_2. The star symbols indicate the materials and the corresponding parameters used in our work. The parameters of our materials are the following. PEDOT:PSS*: volumetric capacitance C_{V} = 43 F cm^{-3}, hole mobility μ = 1.02 cm^2 V^{-1} s^{-1}, threshold voltage V_{TH} = 0.477 V. p(g2T-TT)*: C_{V} = 241 F cm^{-3}, μ = 0.13 cm^2 V^{-1} s^{-1}, and V_{TH} = -0.23 V. The performance estimated with the state-of-art materials are calculated by substituting the material parameters of transistor T_2 with those of the material under investigation. The parameters of the materials investigated (triangle symbols) are taken from Refs.[28–32], and are the following. p(g2T-TT): C_{V} = 241 F cm^{-3}, μ = 0.94 cm^2 V^{-1} s^{-1}, and V_{TH} = -0.2 V. p(g2T-T): C_{V} = 220 F cm^{-3}, μ = 0.28 cm^2 V^{-1} s^{-1}, and V_{TH} = 0 V. p(gBDT-g2T): C_{V} = 77 F cm^{-3}, μ = 0.018 cm^2 V^{-1} s^{-1}, and V_{TH} = -0.55 V.

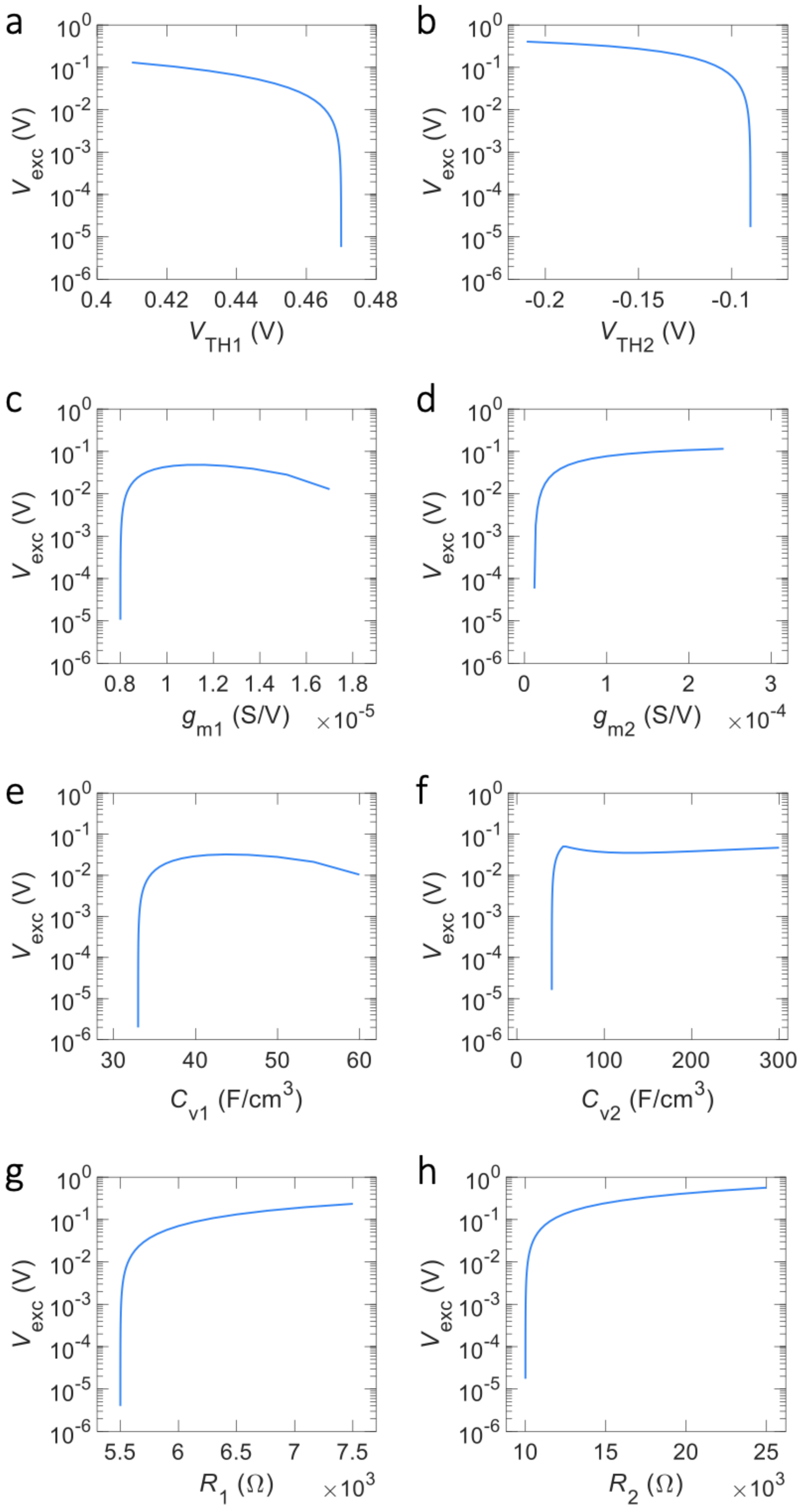

Supplementary Figure 19 | Excitability as a function of OAN parameters. Investigation of the excitability as a function of as a function of OAN parameters of **a** threshold voltage of T_1, V_{TH1}, **b** threshold voltage of T_2, V_{TH2}, **c** normalized transconductance of T_1, g_{m1}, **d** normalized transconductance of T_2, g_{m2}, **e** volumetric capacitance of T_1, C_{V1}, **f** volumetric capacitance of T_2, C_{V2}, **g** resistance of resistor R_1, and **h** resistance of resistor R_2.

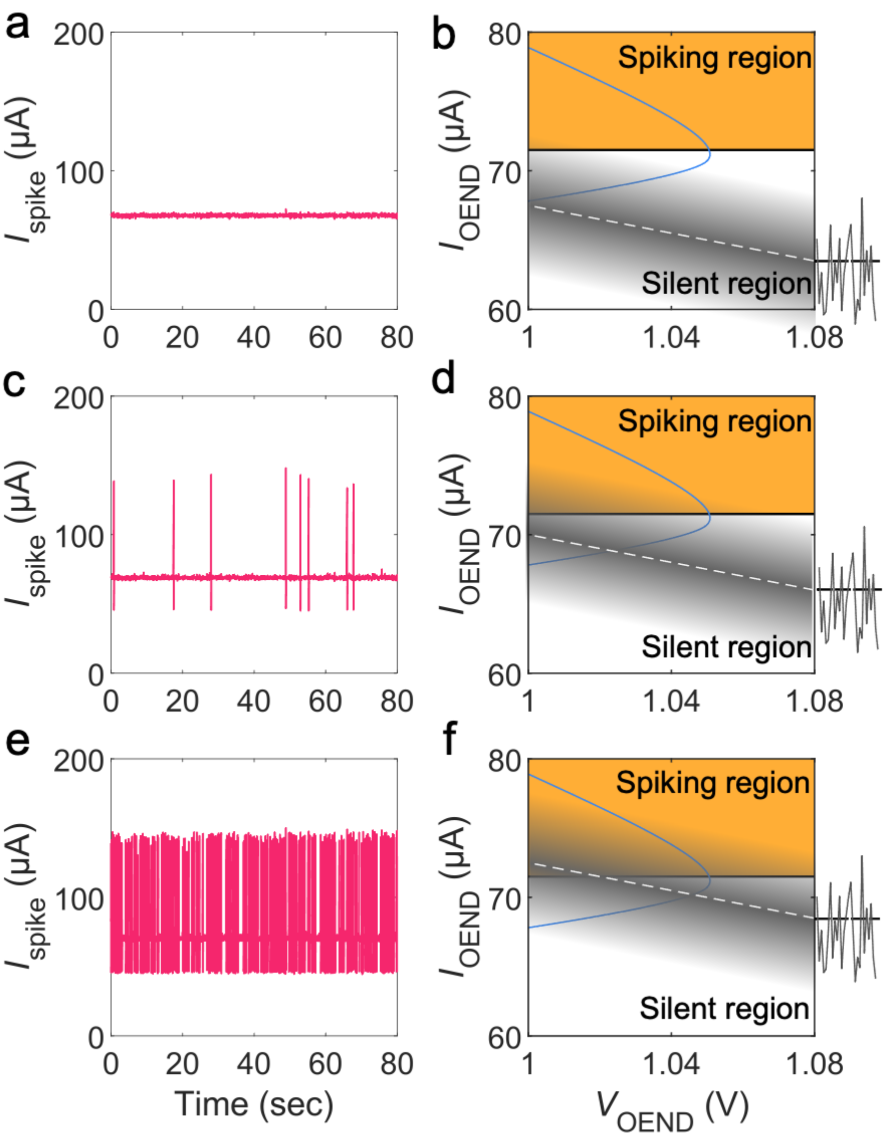

Supplementary Figure 20 | Noise-induced activity. White noise signal is injected into the electrolyte. The noise modulates the position of the load-line as a function of time (shade area) and elicits interrupted spikes. **a,b** The OAN is silent and because the noise is below the excitability threshold. **c,d** Increasing the excitability few random spikes are obtained in the cases the amplitude of noise is above the excitability threshold. **e,f** Further increasing the excitability nearly tonic firing is obtained.

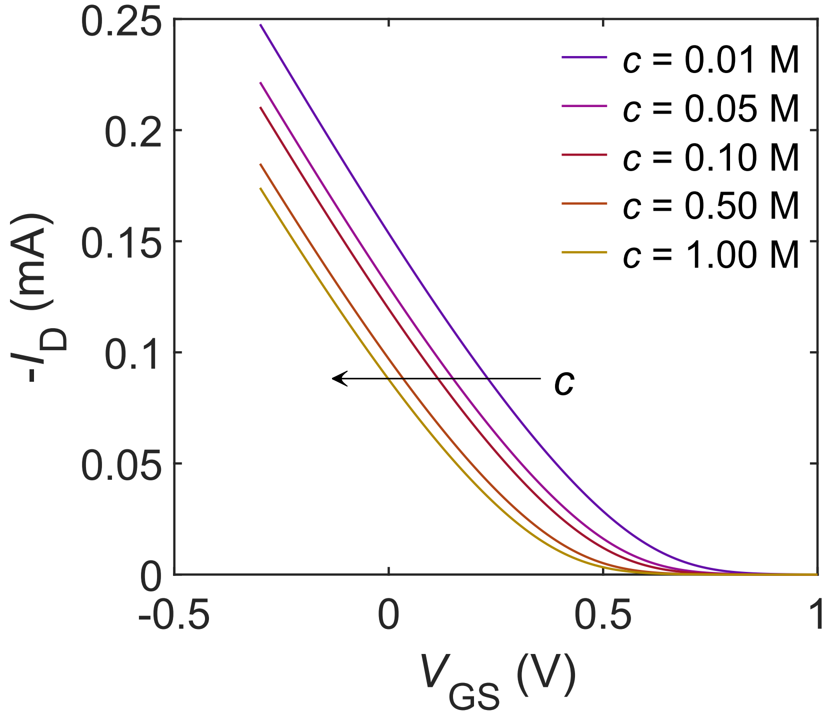

Supplementary Figure 21 | Impact of ion concentration on OECT. Transfer characteristics of OECT T_1 at various ion concentrations. Increasing the ion concentration, the device threshold voltage V_{TH1} charges, and the electrical characteristics shift lo lower gate voltages.

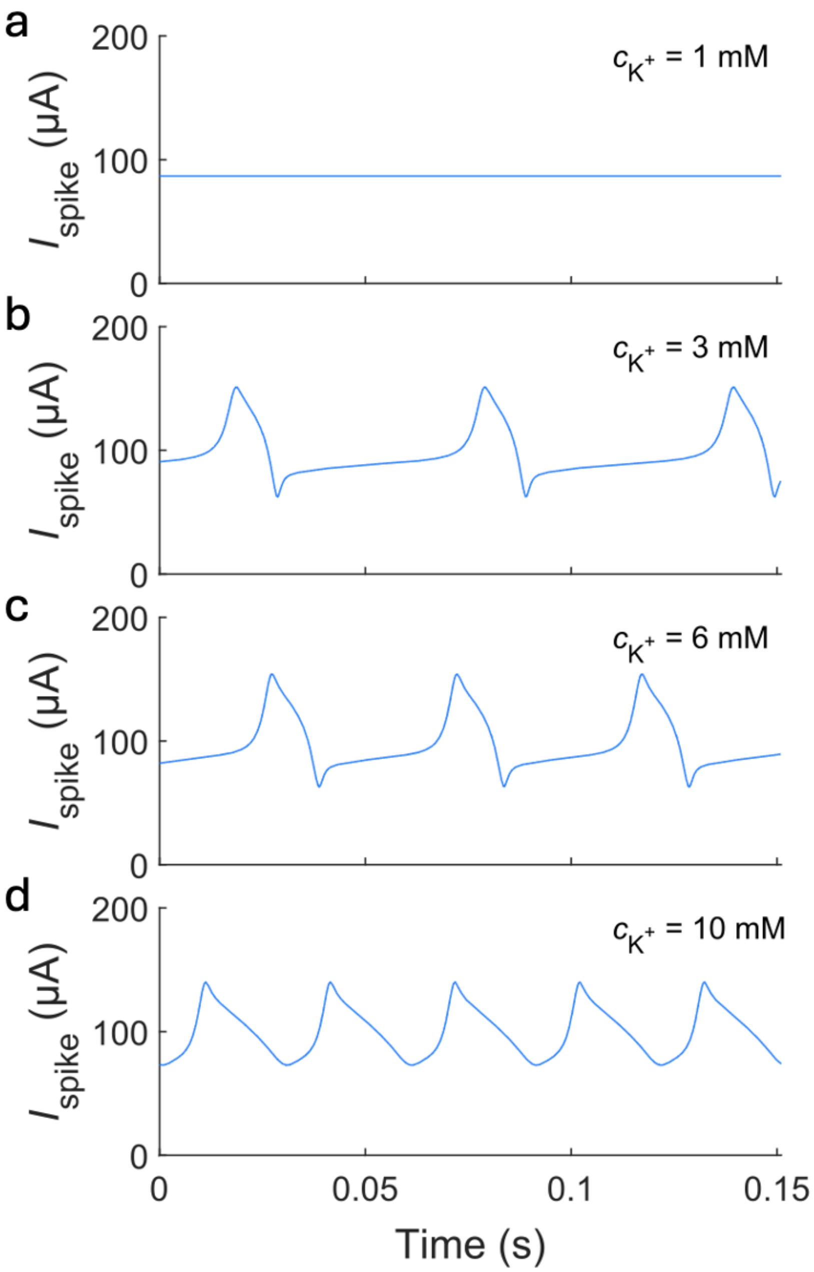

Supplementary Figure 22 | OAN spiking at various K^+ concentrations. Transient numerical simulations. **a** The OAN is silent because the ion concentration c_{K^+} is too low. **b** Increasing the ion concentration, the OAN overcomes the firing threshold. **c, d** Further increasing the ion concentration in the physiological range, the spiking frequency increases.

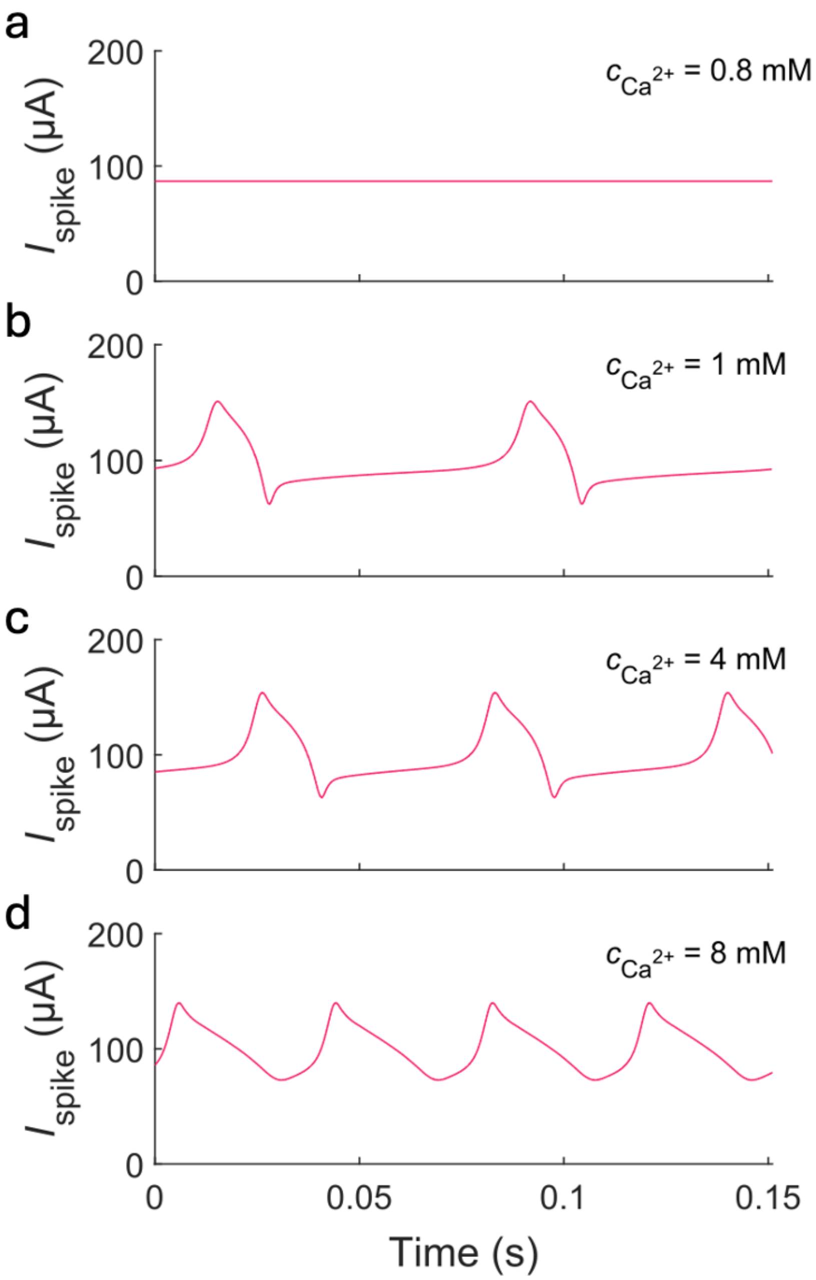

Supplementary Figure 23 | OAN spiking at various Ca^{2+} concentrations. Transient numerical simulations. **a** The OAN is silent, because the ion concentration $c_{Ca^{2+}}$ is too low. **b** Increasing the ion concentration, the OAN overcomes the firing threshold. **c, d** Further increasing the ion concentration in the physiological range, the spiking frequency increases.

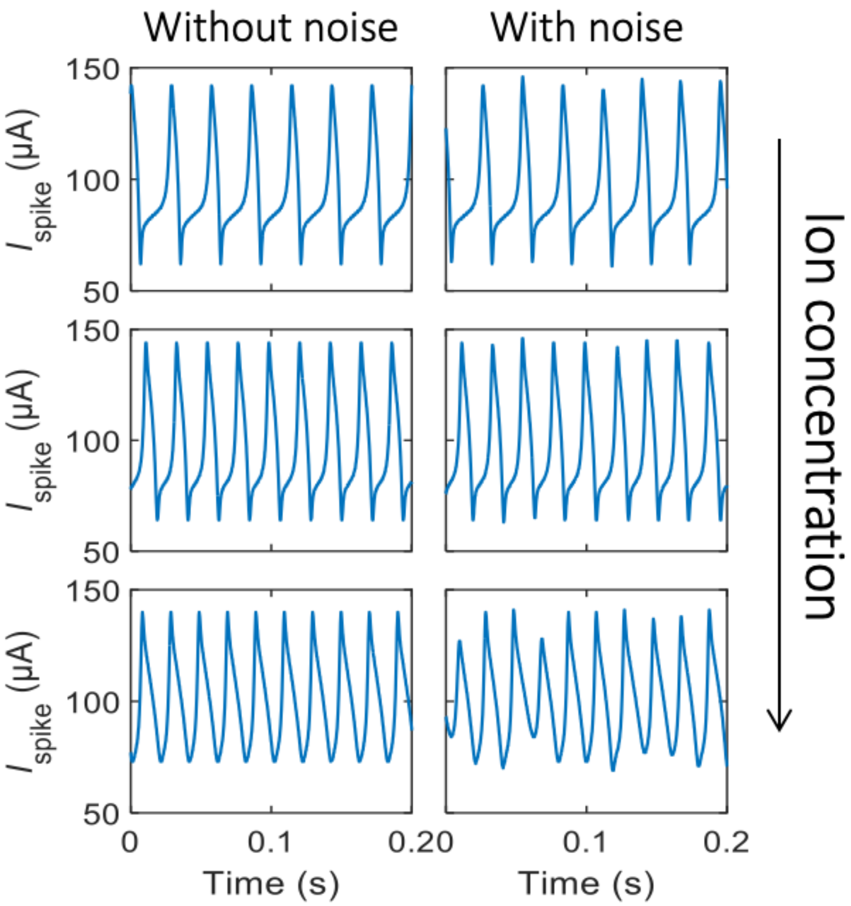

Supplementary Figure 24 | Neuromorphic ion sensing. Spiking current as a function of the ion concentration (c = 3·10^{-3} M, 9.5·10^{-3} M, 30·10^{-3} M) without and with noise injected in the electrolyte of the OAN.

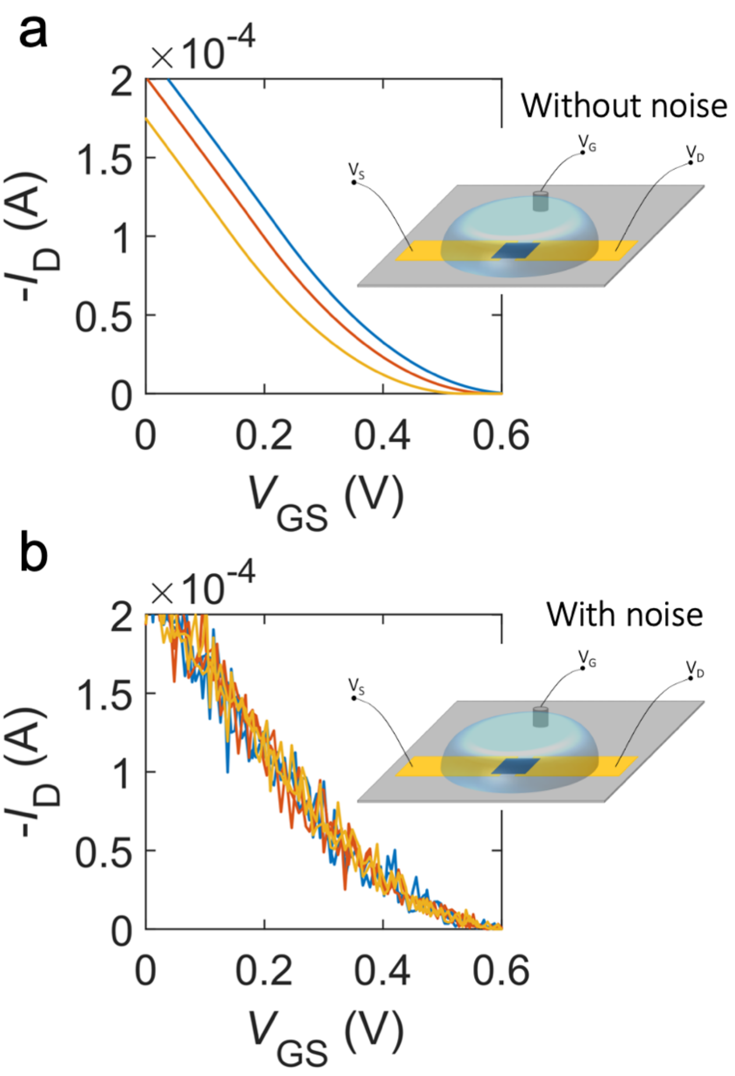

Supplementary Figure 25 | OECT-based ion sensing. OECT drain current as function of the ion concentration ($c = 3 \cdot 10^{-3}$ M, $9.5 \cdot 10^{-3}$ M, $30 \cdot 10^{-3}$ M) **a** without noise and **b** with noise.

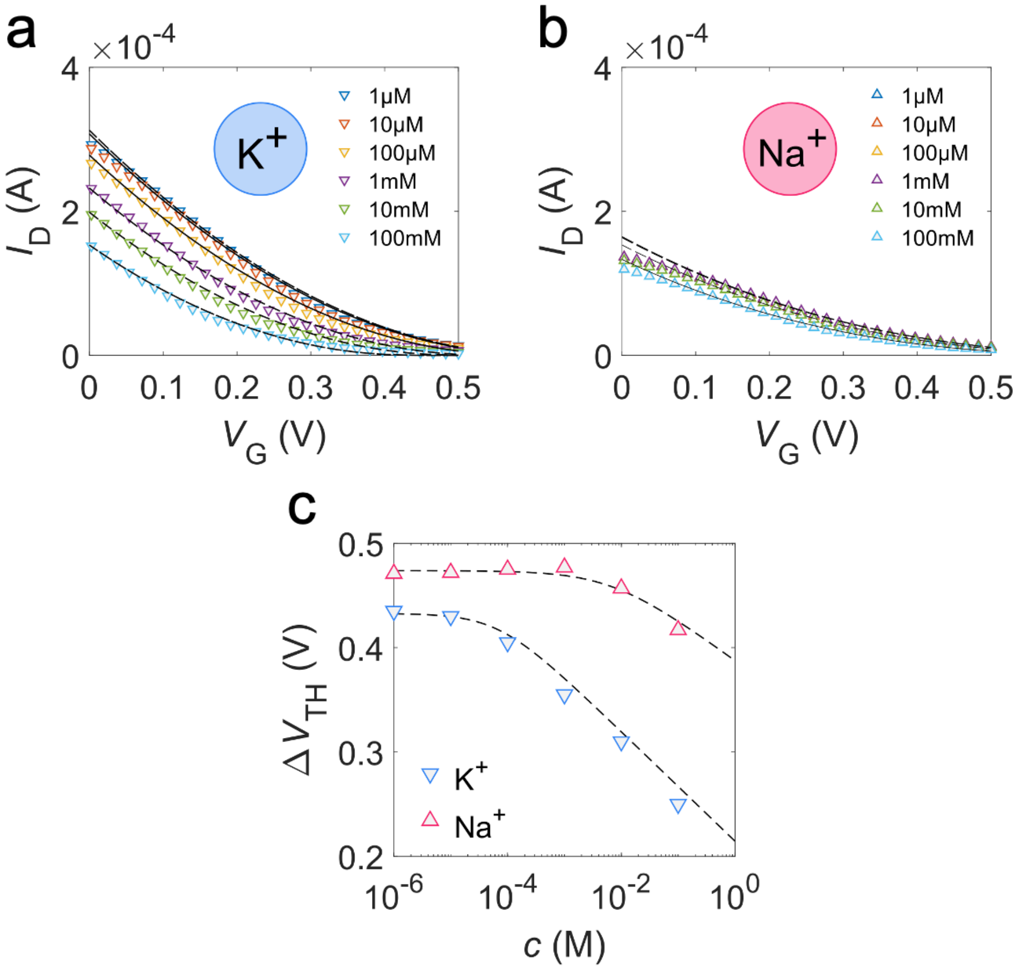

Supplementary Figure 26 | Ion selective OECTs. Measured transfer characteristics (symbols) at various **a** potassium (K^+) and **b** sodium (Na^+) concentration. V_{D} = -0.1V. A K^+-selective ISM is used. Full lines are calculated with the drain-current model in Supplementary Note 1. **c** Threshold voltage variation $\Delta V_{\mathrm{TH}} = V_{\mathrm{TH}}(c) - V_{\mathrm{TH}}(c_{\mathrm{min}})$ as a function of c_{K^+} and c_{Na^+} concentration. K^+ are the target ions and Na^+ are the interfering ions. Dashed line is calculated with Equation (10) in Supplementary Note 1. The ISM model parameters extracted from the measurements are the following: s_{i} = -52.2 mV dec^{-1}, s_{j} = -40 mV dec^{-1}, α = 0.164, $V^{O}_{\mathrm{K}^+}$ = 0.432 V, and $V^{O}_{\mathrm{Na}^+}$ = 0.474 V.

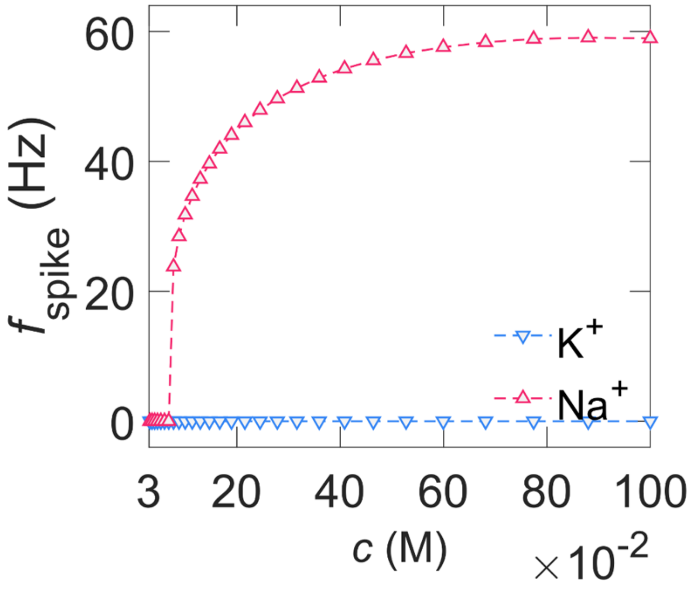

Supplementary Figure 27 | Ion-selective spiking. Spiking frequency f_{spike} as a function of selected Na^+ concentration c. The OAN is not spiking when the concentration of interfering ions (K^+) is varied.

Supplementary Note 4 | Case V_{ON} = V_{OFF}

The condition $V_{\mathrm{ON}} = V_{\mathrm{OFF}}$ can be found by equating Supplementary Equations (16)-(18), and results:

$$R_2 g_{\mathrm{m1}}\left[V_{\mathrm{TH1}}^{\gamma_1} - (V_{\mathrm{TH1}} + V_{\mathrm{TH2}})^{\gamma_1}\right] - V_{\mathrm{TH2}} = \sqrt[\gamma_2]{\frac{V_{\mathrm{TH1}}}{g_{\mathrm{m2}} R_1}} + V_{\mathrm{TH1}} - V_{\mathrm{TH2}} \qquad (27)$$

By solving for the V_{TH1}, we obtain the implicit function:

$$V_{\mathrm{TH1}} = R_2 g_{\mathrm{m1}}\left[V_{\mathrm{TH1}}^{\gamma_1} - (V_{\mathrm{TH1}} + V_{\mathrm{TH2}})^{\gamma_1}\right] - \sqrt[\gamma_2]{\frac{V_{\mathrm{TH1}}}{g_{\mathrm{m2}} R_1}} \qquad (28)$$

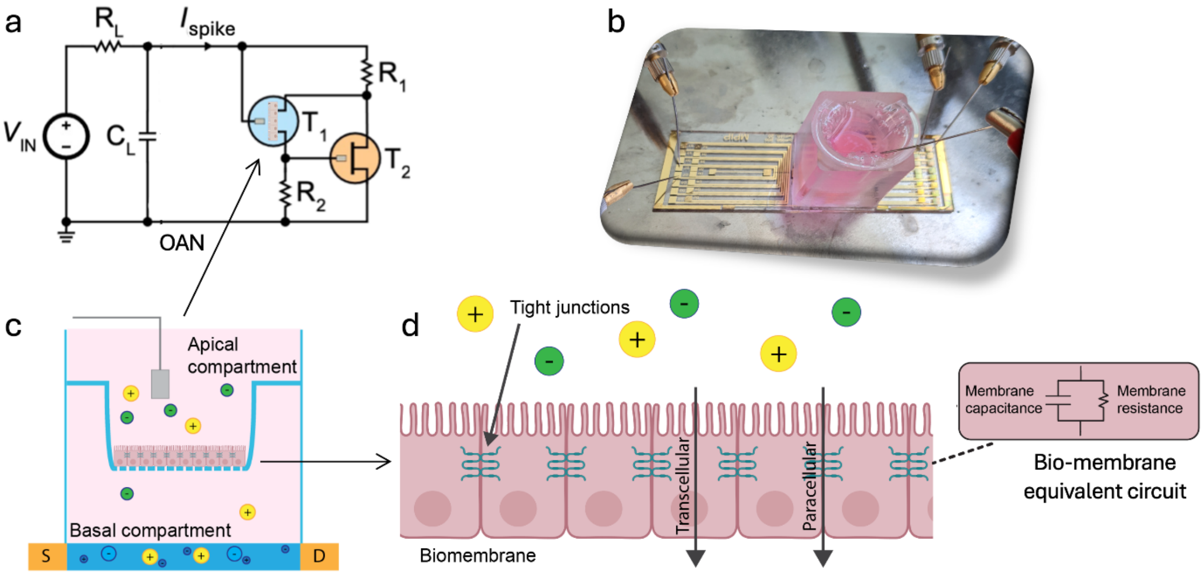

Supplementary Figure 28 | Schematic of the biohybrid OAN. a Circuit diagram of the biohybrid OAN. **b** Photograph of the cellular membrane integrated with the OAN. **c** Simplified cross-section of T_1 with the cellular membrane in the trans-well filter. The gate is inserted in the electrolyte of the apical compartment and the channel is in contact with the electrolyte of the basal compartment. Cell medium is used as electrolyte. **d** Schematic of the epithelial cell layer acting as a physical barrier against ion movement between the basal and apical domains. The cells are interconnected by tight junctions (TJs), forming a barrier that restricts ion flow across the membrane. Ion movement occurs through two potential pathways: the transcellular route (through the cells) and the paracellular route (between the cells). Toxins like hydrogen peroxide (H_2O_2) can disrupt the TJs, impairing the paracellular pathway for ion transport. The equivalent circuit of the bio-membrane is represented by a parallel R_M-C_M circuit, with R_M denoting the transmembrane resistance and C_M representing the membrane capacitance. Toxins such as H_2O_2 can compromise the integrity of the TJs, leading to increased transmembrane ion permeability and a reduction in both R_M and C_M. In a biohybrid neuron initially biased below the firing threshold, the introduction of H_2O_2 disrupts the TJs of the bio-membrane, consequently initiating firing.

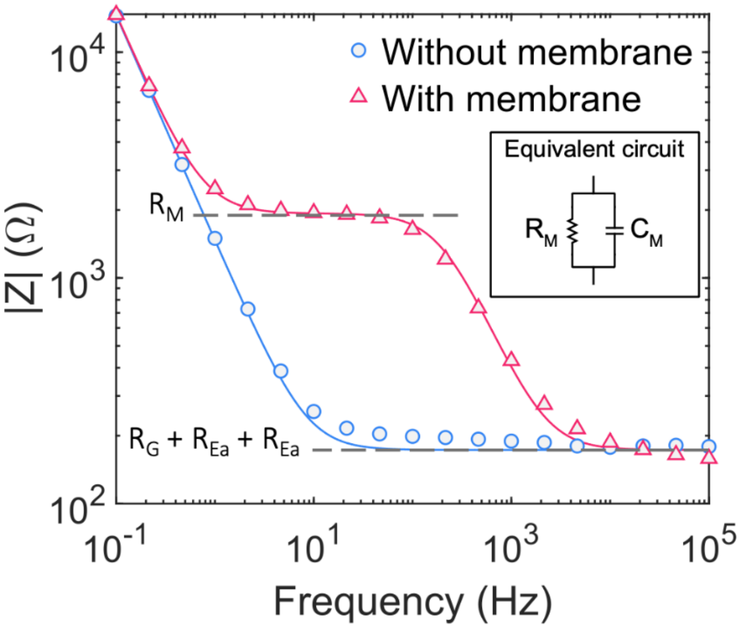

Supplementary Figure 29 | Bio-membrane electrochemical impedance spectroscopy. Measurements (symbols) and model (solid lines) of the biomembrane impedance. Impedance spectrum without the cellular barrier membrane (blue circles) shows a resistor-capacitor (R_B-C_P) circuit, where $R_B = R_G + R_{Ea} + R_{Eb}$, R_G is the gate resistance, R_{Ea} is the apical electrolyte resistance, R_{Eb} is the basal electrolyte resistance and C_P is the capacitance of the OMIEC channel. As a further confirmation, C_P extracted from impedance spectroscopy is normalized to the volume of the PEDOT:PSS, obtaining a volumetric capacitance of 43 F cm^{-3}, in full agreement with the state.[33–35] The membrane equivalent circuit comprises a resistor R_M in parallel with a capacitor C_M. The cellular membrane parameters obtained from the electrochemical impedance spectroscopy are R_M = 1.75 kΩ and C_M = 470 nF. Importantly, we note that the membrane resistance R_M is about one orders of magnitude larger than the background resistance R_B.

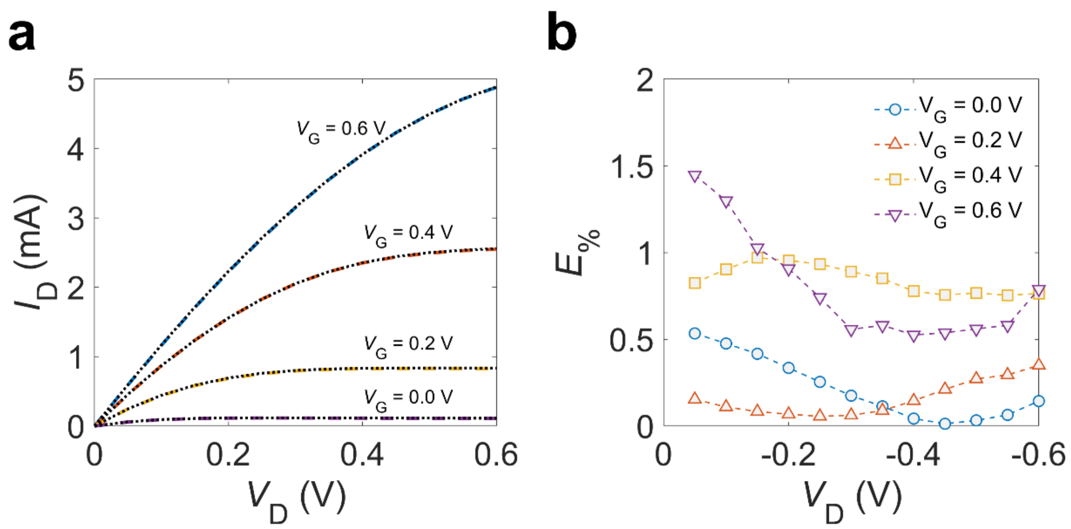

Supplementary Figure 30 | Impact of the toxic compound on the OECT. a Measured output characteristics (I_D-V_D) at various gate voltages V_G in PBS (colour lines) and in PBS with addition of $1 \cdot 10^{-3}$ M H_2O_2 (dotted black lines). **b** $E_{\%} = 100 \cdot (I_{D,PBS} - I_{D,H_2O_2})/I_{D,PBS}$, where $I_{D,PBS}$ is the drain current measured using only PBS as electrolyte and I_{D,H_2O_2} is the drain current measured using PBS with addition of $1 \cdot 10^{-3}$ M H_2O_2. The measurements show that the toxic compound has a negligible impact on the device.

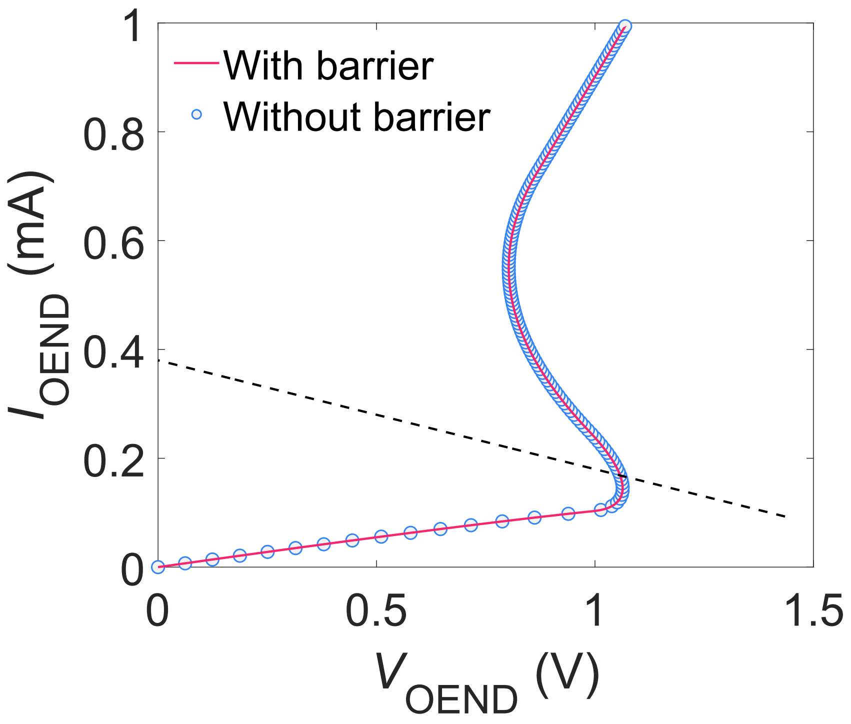

Supplementary Figure 31 | OEND characteristics with and without cellular barrier. OEND electrical characteristic accessed in current mode with (red solid line) and without (blue circles) the cellular barrier. The black dashed line is the load line characteristic. The presence of the barrier does not change the static characteristic of the OEND.

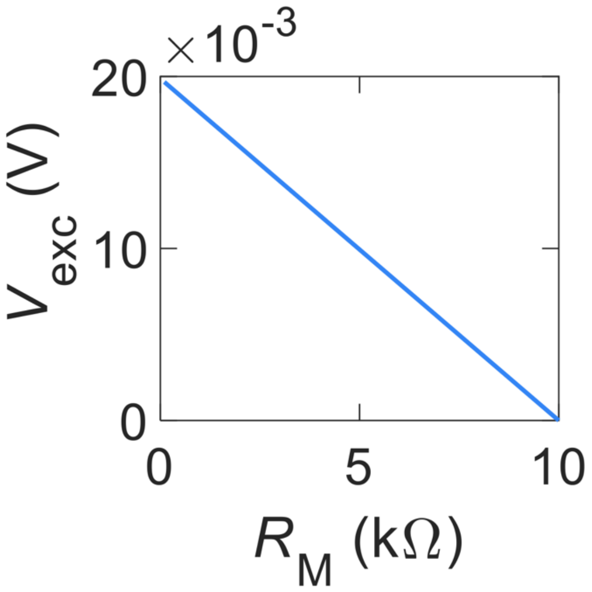

Supplementary Figure 32 | Bio-hybrid excitability modulation. Excitability voltage, V_{exc}, as a function of cellular membrane resistance, R_{M}, which is related to the status of the cellular membrane.

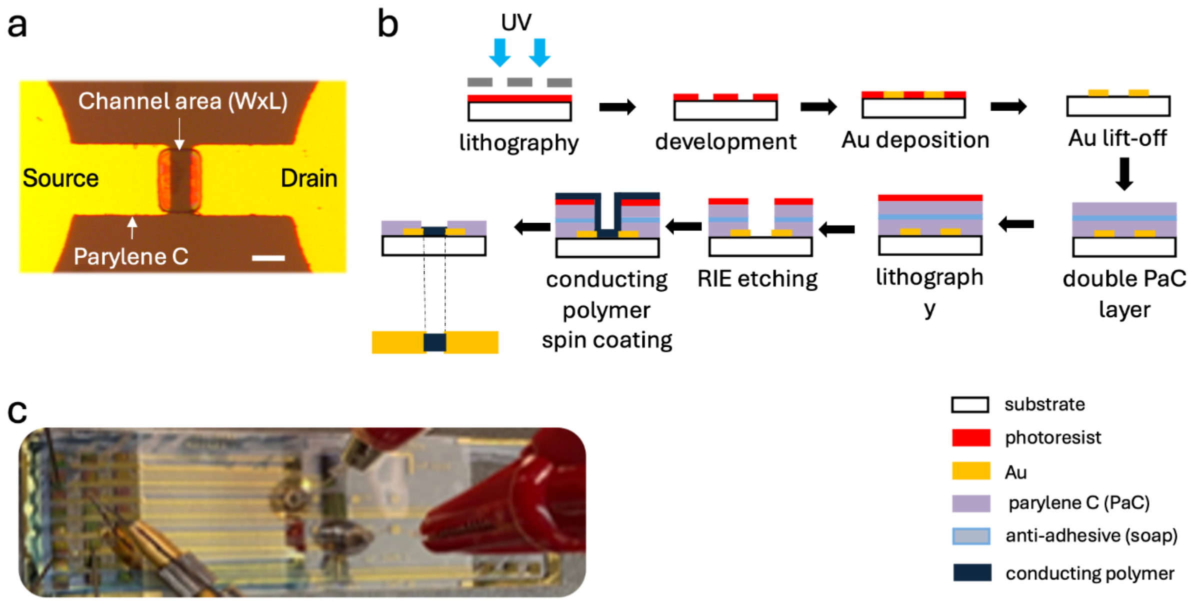

Supplementary Figure 33 | Diagram of device fabrication process. a Photograph of transistor channel with dimensions W = 50 μm, and L = 20 μm. Scale bar length is of 20 μm. **b** Standard microscope glass slides (75 mm × 25 mm) were cleaned in a sonicated bath, first in soap solution (Micro-90 (Sigma-Aldrich)) and then in a 1:1 (vol/vol) solvent mixture of acetone and isopropanol. Source and drain electrodes were made with photolithographically patterned gold (with positive Microposit S1813 photoresist (DOW)) on the cleaned glass slides. A chromium layer was used to improve the adhesion of gold. Two layers of parylene C (SCS Coatings) were deposited. Soap (Micro-90 soap solution, 1% vol/vol in deionized water) was used for separation between the parylene C layers to enable the peel-off of the upper parylene C layer. The lower parylene C layer insulates the gold electrodes. Silane A-174 (γ-methacryloxypropyl trimethoxysilane) from Sigma-Aldrich was added to the lower parylene C layer to enhance adhesion. The channel dimensions of the transistors were defined in the second photolithography step through the positive photoresist AZ 9260 MicroChemicals (Cipec Spécialités). Reactive ion etching (O2/CF4 plasma, 160 W for 16 min with O2 flow rate of 50 s.c.c.m. and CHF3 flow rate of 5 s.c.c.m.) was used to define the transistor channels throughout the photoresist mask. Channel of transistor T_1 is made with the organic mixed ionic–electronic conductor polymer PEDOT:PSS (Clevios PH 1000) mixed with 5.0 wt% ethylene glycol, 0.1 wt% dodecyl benzene sulfonic acid and 1.0 wt% (3-glycidyloxypropyl)trimethoxysilane. Spin coating was used to produce a film in two steps at 1,500 rpm and 650 rpm for 1 min and annealed at 120 °C for 1 min in between. Channel of transistor T_2 is made with the semiconducting polymer p(g2T-TT), dissolved in chloroform (3 mg ml^{-1}) inside a N_2-filled glovebox and spin coated in ambient conditions at 1,000 rpm for 1 min resulting in a thickness of 40 nm. The devices were baked at 60 °C for 1 min. The sacrificial upper parylene C layer was peeled off to confine the polymer to the inside of the channel regions. The devices were subsequently baked at 140 °C for 1 hour. Excess soap was rinsed off with deionized water. **c** Photograph of the OAN device.

Supplementary References

1. Abu-Hassan, K. *et al.* Optimal solid state neurons. *Nature Communications 2019 10:1* **10**, 1–13 (2019).
2. Serb, A. *et al.* Unsupervised learning in probabilistic neural networks with multi-state metal-oxide memristive synapses. *Nat Commun* **7**, 1–9 (2016).
3. Christensen, D. V. *et al.* 2022 roadmap on neuromorphic computing and engineering. *Neuromorphic Computing and Engineering* **2**, 022501 (2022).
4. Cozzolino, O. *et al.* Understanding spreading depression from headache to sudden unexpected death. *Front Neurol* **9**, 333507 (2018).
5. Mulaosmanovic, H., Chicca, E., Bertele, M., Mikolajick, T. & Slesazeck, S. Mimicking biological neurons with a nanoscale ferroelectric transistor. *Nanoscale* **10**, 21755–21763 (2018).
6. Torrejon, J. *et al.* Neuromorphic computing with nanoscale spintronic oscillators. *Nature 2017 547:7664* **547**, 428–431 (2017).
7. Hao, S. *et al.* A Monolayer Leaky Integrate-and-Fire Neuron for 2D Memristive Neuromorphic Networks. *Adv Electron Mater* **6**, 1901335 (2020).
8. Inagaki, T. *et al.* Collective and synchronous dynamics of photonic spiking neurons. *Nature Communications 2021 12:1* **12**, 1–8 (2021).
9. Bohaichuk, S. M. *et al.* Fast Spiking of a Mott VO2-Carbon Nanotube Composite Device. *Nano Lett* **19**, 6751–6755 (2019).
10. Yi, W. *et al.* Biological plausibility and stochasticity in scalable VO2 active memristor neurons. *Nature Communications 2018 9:1* **9**, 1–10 (2018).
11. Zhang, X. *et al.* An artificial spiking afferent nerve based on Mott memristors for neurorobotics. *Nature Communications 2020 11:1* **11**, 1–9 (2020).
12. Park, S. O., Jeong, H., Park, J., Bae, J. & Choi, S. Experimental demonstration of highly reliable dynamic memristor for artificial neuron and neuromorphic computing. *Nature Communications 2022 13:1* **13**, 1–13 (2022).
13. Pickett, M. D., Medeiros-Ribeiro, G. & Williams, R. S. A scalable neuristor built with Mott memristors. *Nature Materials 2013 12:2* **12**, 114–117 (2012).
14. Kumar, S., Strachan, J. P. & Williams, R. S. Chaotic dynamics in nanoscale NbO2 Mott memristors for analogue computing. *Nature 2017 548:7667* **548**, 318–321 (2017).
15. Kumar, S., Williams, R. S. & Wang, Z. Third-order nanocircuit elements for neuromorphic engineering. *Nature 2020 585:7826* **585**, 518–523 (2020).
16. Tee, B. C. K. *et al.* A skin-inspired organic digital mechanoreceptor. *Science (1979)* **350**, 313–316 (2015).
17. Mirshojaeian Hosseini, M. J. *et al.* Organic electronics Axon-Hillock neuromorphic circuit: towards biologically compatible, and physically flexible, integrate-and-fire spiking neural networks. *J Phys D Appl Phys* **54**, 104004 (2020).
18. Harikesh, P. C. *et al.* Organic electrochemical neurons and synapses with ion mediated spiking. *Nature Communications 2022 13:1* **13**, 1–9 (2022).
19. Harikesh, P. C. *et al.* Ion-tunable antiambipolarity in mixed ion–electron conducting polymers enables biorealistic organic electrochemical neurons. *Nature Materials 2023 22:2* **22**, 242–248 (2023).
20. Sarkar, T. *et al.* An organic artificial spiking neuron for in situ neuromorphic sensing and biointerfacing. *Nat Electron* **5**, 774–783 (2022).
21. Friedlein, J. T. *et al.* Optical Measurements Revealing Nonuniform Hole Mobility in Organic Electrochemical Transistors. *Adv Electron Mater* **1**, 1500189 (2015).
22. Bernards, D. A. & Malliaras, G. G. Steady-State and Transient Behavior of Organic Electrochemical Transistors. *Adv Funct Mater* **17**, 3538–3544 (2007).

23. Torricelli, F. *et al.* Transport physics and device modeling of zinc oxide thin-film transistors - Part II: Contact resistance in short channel devices. *IEEE Trans Electron Devices* **58**, 3025–3033 (2011).
24. Romele, P., Ghittorelli, M., Kovács-Vajna, Z. M. & Torricelli, F. Ion buffering and interface charge enable high performance electronics with organic electrochemical transistors. *Nat Commun* **10**, 1–11 (2019).
25. Fakih, I. *et al.* Selective ion sensing with high resolution large area graphene field effect transistor arrays. *Nature Communications 2020 11:1* **11**, 1–12 (2020).
26. Li, Y. *et al.* Ion-Selective Organic Electrochemical Transistors: Recent Progress and Challenges. *Small* **18**, 2107413 (2022).
27. Crespo, G. A. & Bakker, E. Dynamic electrochemistry with ionophore based ion-selective membranes. *RSC Adv* **3**, 25461–25474 (2013).
28. Inal, S., Malliaras, G. G. & Rivnay, J. Benchmarking organic mixed conductors for transistors. *Nature Communications 2017 8:1* **8**, 1–7 (2017).
29. Li, P. & Lei, T. Molecular design strategies for high-performance organic electrochemical transistors. *Journal of Polymer Science* **60**, 377–392 (2022).
30. Giovannitti, A. *et al.* N-type organic electrochemical transistors with stability in water. *Nature Communications 2016 7:1* **7**, 1–10 (2016).
31. Fenoy, G. E., von Bilderling, C., Knoll, W., Azzaroni, O. & Marmisollé, W. A. PEDOT:Tosylate-Polyamine-Based Organic Electrochemical Transistors for High-Performance Bioelectronics. *Adv Electron Mater* **7**, 2100059 (2021).
32. Giovannitti, A. *et al.* Controlling the mode of operation of organic transistors through side-chain engineering. *Proc Natl Acad Sci U S A* **113**, 12017–12022 (2016).
33. Koutsouras, D. A. *et al.* Probing the Impedance of a Biological Tissue with PEDOT:PSS-Coated Metal Electrodes: Effect of Electrode Size on Sensing Efficiency. *Adv Healthc Mater* **8**, 1901215 (2019).
34. Koutsouras, D. A. *et al.* Impedance Spectroscopy of Spin-Cast and Electrochemically Deposited PEDOT:PSS Films on Microfabricated Electrodes with Various Areas. *ChemElectroChem* **4**, 2321–2327 (2017).
35. Rivnay, J. *et al.* High-performance transistors for bioelectronics through tuning of channel thickness. *Sci Adv* **1**, (2015).
